# Integrated Assessment of Silver Nanoparticles on Plant Growth and Cytogenotoxicity Using *Triticum* and *Allium* Bioassays

**DOI:** 10.3390/jox15050147

**Published:** 2025-09-12

**Authors:** Simona Elena Pisculungeanu, Liliana Cristina Soare, Oana Alexandra Luțu, Alina Păunescu, Georgiana Cîrstea, Aurelian Denis Negrea, Codruța Mihaela Dobrescu, Nicoleta Anca Ionescu (Șuțan)

**Affiliations:** 1 Department of Natural Sciences, Pitesti University Centre, National University of Science and Technology Politehnica Bucharest, 1st Targu din Vale Str., 110040 Pitesti, Romania; pisculungeanue@gmail.com (S.E.P.); oana.draghiceanu@upb.ro (O.A.L.); alina.paunescu@upb.ro (A.P.); codruta.dobrescu@upb.ro (C.M.D.); nicoleta_anca.sutan@upb.ro (N.A.I.); 2 Regional Research and Development Center for Innovative Materials, Processes and Products for the Automotive Industry (CRC&D-Auto), Pitesti University Center, National University of Science and Technology Politehnica Bucharest, 1st Targu din Vale Str., 110040 Pitesti, Romania; georgiana.cirstea93@upb.ro (G.C.); aurelian.negrea@upb.ro (A.D.N.)

**Keywords:** silver nanoparticles, cytogenotoxicity, mitotic index, phytotoxicity, plant bioassay, environmental safety

## Abstract

The production and extensive use of silver nanoparticles (AgNPs) in various fields necessitate thorough testing, not only in terms of their potential applications but also regarding the effects they induce on various organisms. In addition, nanoparticles generated from various anthropogenic activities, which reach or are formed in the atmosphere, have a significant impact on the health of humans and other living organisms. Recent research indicates that the effects produced by these nanoparticles are dependent on their size and applied dose. In this context, the present study aimed to evaluate the physiological, biochemical and cytogenotoxic effects induced by different doses of AgNPs compared to positive and negative controls in *Triticum aestivum* L. and *Allium cepa* L. A significant stimulatory effect of the treatment performed with the solution of AgNPs with a size of 20 nm, at the lowest concentration (0.02 µg mL^−1^), in the two tested species, was obtained. The growth and weight of the seedling were significantly increased, and the mitotic index was also elevated. Additionally, this treatment variant showed the lowest percentage of chromosomal aberrations. No significant differences were observed in cell viability, total polyphenol content, proline levels, or assimilatory pigment concentrations compared to the control. Our findings show that AgNPs may exert stimulatory effects, whether significant or not, on certain physiological and biochemical parameters. However, they also interfere with cell cycle regulation and genomic stability, raising concerns regarding their environmental and biological safety. The *Allium* test proved to be an effective method for detecting nanoparticle-induced genotoxicity and can be recommended as a preliminary screening assay in nanoparticle safety evaluations.

## 1. Introduction

The widespread use of nanomaterials, particularly silver nanoparticles (AgNPs), in consumer products, food packaging, and medical applications has raised significant concerns regarding their biological safety. Moreover, nanoparticles (NPs) originating from various anthropogenic activities and subsequently released or formed in the atmosphere exert a substantial impact on human health and the well-being of other living organisms [1].

Due to their small size and high reactivity, silver nanoparticles (AgNPs) can interact with cellular structures, potentially inducing oxidative stress, DNA damage, and disruption of mitotic processes [2,3]. These alterations at the cellular and molecular levels may also manifest in physiological and biochemical responses, affecting plant growth and various stress-related biochemical parameters, such as proline, polyphenol, and chlorophyll pigment contents, which serve as biomarkers of stress.

Recent studies indicate that plant responses to AgNPs vary depending on species, cultivar, ontogenetic stage, and nanoparticle characteristics such as size, applied dose, and exposure duration. The AgNPs tested by Guzmán-Báez et al. [4] stimulated early growth and biomass production in tomato plants and improved their nutrient status. Root growth, root number, and both fresh and dry shoot biomass, as well as fresh root biomass, were significantly increased following AgNP application. Similarly, treatment with 20 mg L^−1^ AgNPs enhanced shoot number, shoot length, and both fresh and dry biomass in *Maerua oblongifolia* plants [5]. In wheat, exposure to 20 and 40 ppm AgNPs led to increased fresh and dry weight (d.w.) of the plants [6].

Smaller AgNPs (10 and 20 nm) have been shown to reduce root growth and fresh weight (f.w.) in *T. aestivum*, without inducing significant changes in d.w. [7]. AgNPs (20 nm, 5–10 ppm) were also found to alter the expression of proteins involved in membrane integrity and cellular processes [8]. The inhibitory effect of AgNPs on root elongation is species-dependent [9]; for instance, in wheat, root length was reduced by 26.39% compared to the control, whereas in tomato, the same concentration of AgNPs (100 mg L^−1^) caused a considerably lower reduction of 7.30% [10].

Free proline content has been recognized as a reliable indicator of stress in plants [11]. Exposure to AgNPs can alter proline levels [12,13]. Variations in photosynthetic pigment and polyphenol contents have also been reported in recent studies [14,15,16,17].

These AgNP-induced changes can be quantified using various standardized assays, such as the *Triticum* test, Evans blue test, or *Allium* test. The *Triticum* assay evaluates plant growth parameters (axial organ elongation and biomass), physiobiological indicators (chlorophyll and carotenoid content), and biochemical markers (proline and phenolic content) under diverse abiotic or biotic stress conditions [18]. The Evans blue test provides a rapid assessment of plant cell membrane integrity, thereby indicating cell viability under different treatments [19].

The *Allium cepa* L. assay is a well-established model widely employed to assess the cytotoxic and genotoxic effects of environmental contaminants. This assay offers several advantages, including high sensitivity to clastogenic and aneugenic agents, ease of root meristem observation, and strong correlation with mammalian test systems [20].

In this context, the present study aimed to evaluate the physiological, biochemical, and cytogenotoxic effects induced by varying doses of AgNPs, compared to positive and negative controls, by analyzing root and stem growth, fresh and d.w., assimilatory pigments, and polyphenol and proline content in *T. aestivum* seedlings, as well as cell viability, mitotic index, phase distribution, and the frequency and types of chromosomal aberrations in the root meristem of *A. cepa* L.

## 2. Materials and Methods

### 2.1. Nanoparticles Used

For the proposed research, a standard AgNPs colloidal solution (nominal size 20 nm, concentration 0.02 mg·mL^−1^, with maximum absorption at 405 nm), stabilized with 2 mM sodium citrate, was obtained from Thermo Scientific (Ward Hill, MA, USA). To verify the size of the NPs, the stock solution was characterized by bright field scanning transmission electron microscopy (BFSTEM) using HITACHI SU8230 microscope (Hitachi, Tokyo, Japan) in the Advanced Materials laboratory (Regional Center of Research & Development for Materials, Processes and Innovative Products Dedicated to the Automotive Industry—CRCD-Auto). We used AgNps with a size of 20 nm for their ability to be transported and translocated via apoplastic route in the plant, symplastic transport not being excluded. Citrate (˂0.1% *w*/*w*) is a metabolite produced by plants, exudate from roots, frequently used as a buffer in research and which can modify the toxicity of AgNPs [21].

### 2.2. Experimental Variants

To evaluate the influence of AgNPs on the growth of *T. aestivum* seedlings, as well as on the content of assimilatory pigments, proline, total polyphenols and cell viability, the experimental variants were detailed in Table 1. For each experimental variant, 10 seeds were used, and each treatment was performed in triplicate.

All experimental variants described in Table 1 were applied consistently across all bioassays in this study, including *T. aestivum* seedling growth and biochemical assays, Evans Blue cell viability test, and *A. cepa* cytogenotoxicity assay. These variants comprised a negative control (distilled water), solvent control (2 mM sodium citrate), and three concentrations of AgNPs (N1, N2, N3) diluted in 2 mM sodium citrate. These concentrations are in line with the incremental increase in soil AgNP concentrations predicted in the most recent modeling [22].

### 2.3. Wheat Exposure to the Test Solutions

To evaluate the influence of AgNPs on the growth of *T. aestivum* seedlings, as well as on the content of assimilatory pigments, proline, total polyphenols, and cell viability, for each experimental variant, 10 seeds were used, and each treatment was performed in triplicate. This step was carried out at the seed stage, following a previously tested method [23]. Wheat caryopses of the Trivale variety (provided from Agricultural Research and Development Station Pitești, Argeș county, Albota, Romania) were hydrated in distilled water for one hour and subsequently immersed for one hour in the test solutions. They were germinated in Petri dishes (Borosilicate Glass 90 mm × 15 mm, Novarli, Benešov, Czech Republic), on filter paper (Whatman, Ø 90 mm, Cole-Parmer, Vernon Hills, IL, USA), periodically hydrated with distilled water and maintained at room temperature (25 ± 2 °C), with 16/8 h photoperiod [21]. The Petri dishes were rearranged every day to ensure uniform light exposure.

#### 2.3.1. Determination of Axial Organs Growth and Weight of Wheat Seedlings

Measurement of axial organs length (roots and stems) was performed after 5 days from the initiation of the experiment. The f.w. and the d.w. of the seedlings was determined by weighing on a balance (RADWAG WTC 600) (Radom, Poland). The d.w. of the biological material was obtained by oven (Binder ED 53, Tuttlingen, Germany) drying at 80 °C, until a constant weight was reached. The results obtained are expressed as mean ± standard deviation (SE).

#### 2.3.2. Determination of the Content of Assimilatory Pigments, Polyphenols and Proline

The evaluation of these parameters was carried out from the leaves of *T. aestivum*. The seedlings were obtained according to the above methodology, being transferred from Petri dishes to the Kekkilä professional DSM 3 W peat and hydrated periodically. For the quantification of biological markers in plant leaves, the samples were collected after 21 days from the initiation of the experiment (Figure S2). This time is necessary to ensure a sufficient quantity of leaves in accordance with the working methodologies. In addition, recent research has shown that AgNPs reach the root vascular tissues after 17 days of exposure and are subsequently transported to the rest of the plant. The investigation of chronic toxicity is of interest to evaluate the mechanisms of compensation for stress induced by AgNPs [21].

For the quantitative determination of the assimilatory pigments (expressed as mg g^−1^ f.w.), we used the spectrophotometric method and Holm’s formulas [24]. The total content of phenols, expressed as % gallic acid equivalents (G.A.E., f.w.^−1^), was established with the Singleton and Rossi (1965) [25] method modified by Orțan et al. (2015) [26] and the content of proline (expressed as µmoles g^−1^ f.w.) according to the method described by Bates et al. (1973) [27]. The determinations were performed on a UV-Vis T70+ spectrophotometer (PG Instruments, Lutterworth, UK).

### 2.4. The Assessment of Cell Viability and Cytogenotoxic Effects

The evaluation of cell viability and cytogenotoxic effects was conducted using *A cepa* root meristems as the biological model. The analysis followed established protocols, specifically the Evans Blue assay for cell viability and the *Allium* test for assessing cytogenotoxicity.

#### 2.4.1. The Evans Blue Assay

Cell viability was assessed following the protocol described by Vijayaraghavareddy et al. [19] with minor modifications. After the experimental treatment, ten roots were randomly selected from each onion bulb. The roots were immersed for 15 min in 2 mL of 0.25% Evans Blue solution, then rinsed thoroughly with distilled water to remove excess dye. Subsequently, the roots were kept in distilled water overnight at room temperature. The apical 5 mm segments of the roots were excised the following day and placed in 2 mL of 1% sodium dodecyl sulfate (SDS) aqueous solution, then incubated in a water bath at 50 °C for one hour. The absorbance of the extracted dye was measured at 600 nm using a UV-Vis T70+ spectrophotometer (PG Instruments) for quantitative determination of dye uptake.

#### 2.4.2. The *Allium* Assay

Onion bulbs approximately 4 cm in diameter, grown under organic conditions (no fertilizers or pesticides) and meeting phytosanitary standards, were used. The outer cataphylls were carefully removed to expose the root primordia. Bulbs were placed in 30 mL containers with the discoidal stem immersed in distilled water to induce root growth. Rooted bulbs were exposed to different treatments for a total duration of 72 h. The initial 48 h period involved exposure to distilled water to induce root growth, followed by static exposure for 24 or 48 h to the following test solutions: N1 (N1 24H, N1 48H), N2 (N2 24H, N2 48H), N3 (N3 24H, N3 48H). Distilled water (DW24H, DW48H) was used as negative control, sodium citrate 2 mM (SC24H, SC48H) as solvent control, and methyl methane sulfonate (MMS) 2 mM as positive control. After treatment, root tips were harvested and fixed in Farmer’s solution (ethanol:acetic acid, *v*/*v*, 3:1), hydrolyzed with 1N HCl at 60 °C for 5 min, stained with 1% aceto-orcein, and squashed on slides. A minimum of 3000 cells per treatment were analyzed (Olympus CX-31 microscope, Olympus Corporation, Tokyo, Japan) to determine mitotic index (MI) as number of dividing cells/total cells × 100 [28], mitotic phase distribution (prophase, metaphase, anaphase, telophase) and frequency of chromosomal aberrations.

### 2.5. Statistical Analysis

All results are presented as the mean ± standard error (SE) of at least three independent experiments. Data were subjected to statistical analysis using one-way analysis of variance (ANOVA) to assess the significance of differences among treatment groups. Duncan’s multiple range test was applied as a post hoc comparison to determine statistically distinct means. Differences were considered statistically significant at *p* ≤ 0.05. Robustness of the results was further assessed by applying a bootstrap procedure (simple resampling method, 1000 samples, 95% percentile confidence intervals). Post hoc multiple comparisons were performed using Bonferroni and Dunnett’s tests were applied to control for Type I error and for comparisons with the negative control group, at a significance threshold of α = 0.05. Pearson correlation coefficients (r) were calculated to evaluate the strength and direction of associations between quantitative variables, with 95% confidence intervals reported. All statistical analyses were conducted in SPSS (IBM SPSS Statistics, version 27.0.1).

## 3. Results

### 3.1. Nanoparticle Size Verification

**Figure 1:**
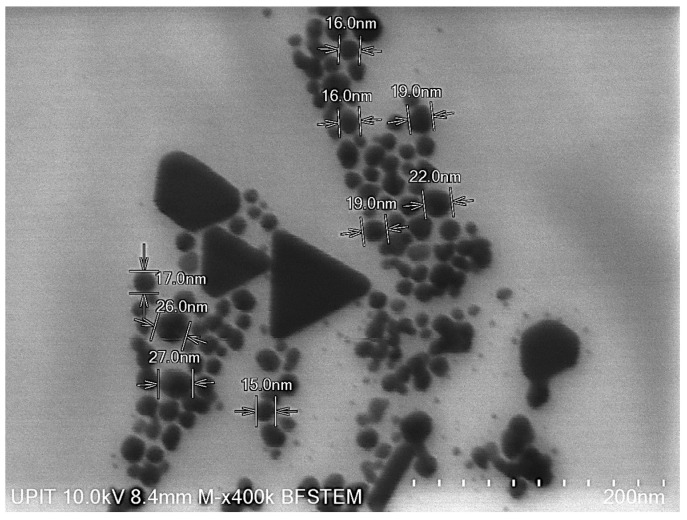
AgNPs dispersion and dimensional analysis performed by BF-STEM.

The analysis revealed an average size of the AgNPs of 19.66 nm, thus confirming the specifications of the standard used (Figure 1). Infrequently, in microscopic sample appear larger triangular and polygonal forms that correspond to agglomeration of AgNPs, which is consistent with observations reported in the literature [29,30] and can occur even in standard reference materials. This forms not appear in all samples (Figure S1. AgNPs dispersion and form analysis performed by BF-STEM).

### 3.2. The Effects Induced by Silver Nanoparticles on Axial Organ Growth and Seedlings Weight of Triticum aestivum L.

**Figure 2:**
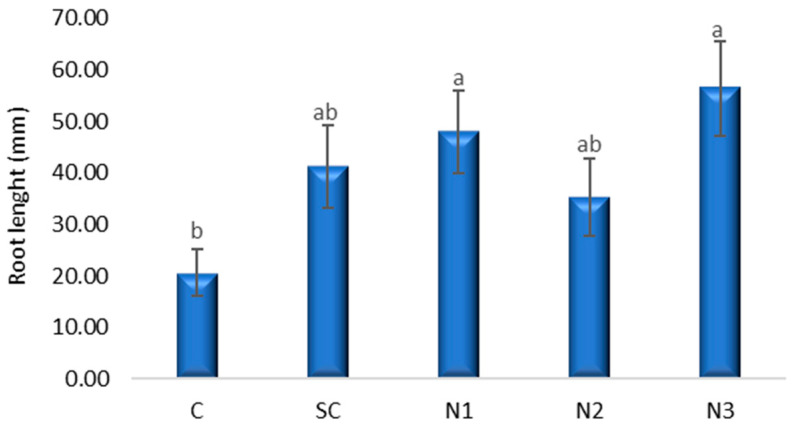
The influence of the AgNPs (20 nm, 0.02 mg/mL dispersed in a 2 mM sodium citrate solution, λmax: 405 nm) on root growth in *T. aestivum* seedlings, 5 days after exposure; (a, b: the interpretation of the significance of the differences by means of the Duncan test, *N* = 15, df = 4, *p* < 0.05).

Root development was stimulated by the presence of AgNPs in all treatment variants (N1, N2, N3), with values exceeding those of the control group (Figure 2). Additionally, SC exhibited a positive effect on root growth that did not differ significantly from the control (*p* = 0.175, 95% CI [−5.9391, 47.2724]). Notably, the average root length at the lowest AgNP concentration (N3) was approximately three times greater than that of the control. Post hoc Bonferroni comparisons indicated that variant N3 had a significantly greater root length than C (*p* = 0.011), while other comparisons were not statistically significant. Using Dunnett’s test with C as the reference, root biomass was significantly increased by N1 (*p* = 0.041, 95% CI [0.7942, 54.0057]) and N3 (*p* = 0.004, 95% CI [9.2942, 62.5057]) relative to the control.

**Figure 3:**
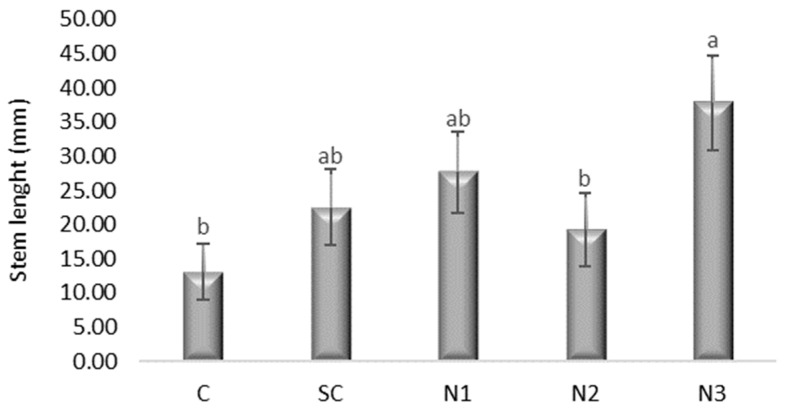
The influence of the AgNPs (20 nm, 0.02 mg/mL dispersed in a 2 mM sodium citrate solution, λmax: 405 nm) on stem growth in *T. aestivum* seedlings, 5 days after exposure; (a, b: the interpretation of the significance of the differences by means of the Duncan test, *N* = 15, df = 4, *p* < 0.05).

ANOVA revealed a significant effect of treatment on stem length (Figure 3) (*F*(4, 145) = 2.701, *p* = 0.033). Bonferroni multiple comparisons indicated that N3 significantly reduced stem length (*p* = 0.025, 95% CI [1.8497, 47.5502]). Dunnett’s test confirmed that N3 significantly increased stem length relative to the control (mean difference = 24.70, *p* = 0.009). No other treatments, including SC, showed significant differences compared to the control (Table S1).

**Figure 4:**
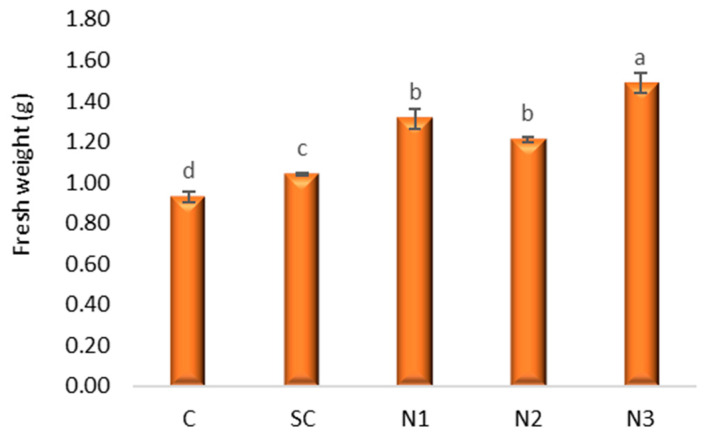
The influence of the AgNPs (20 nm, 0.02 mg/mL dispersed in a 2 mM sodium citrate solution, λmax: 405 nm) on fresh weight in *T. aestivum* L. seedlings, 5 days after exposure; (a–d: the interpretation of the significance of the differences by means of the Duncan test, *N* = 15, df = 4, *p* < 0.05).

**Figure 5:**
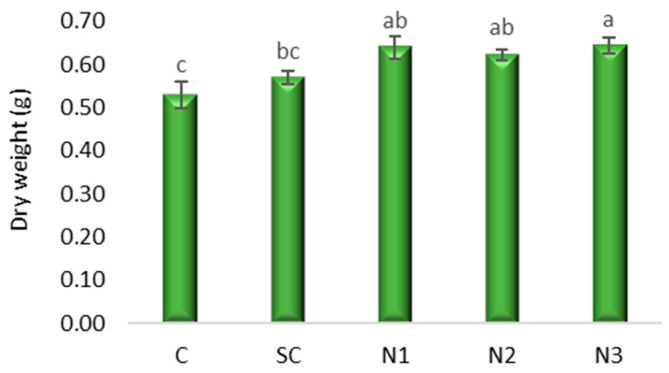
The influence of the AgNPs (20 nm, 0.02 mg/mL dispersed in a 2 mM sodium citrate solution, λmax: 405 nm) on dry weight in *T. aestivum* seedlings, 5 days after exposure; (a–c: the interpretation of the significance of the differences by means of the Duncan test, *N* = 15, df = 4, *p* < 0.05).

The one-way ANOVA indicated significant differences in f.w. among treatments (*F*(4, 10) = 40.85, *p* < 0.001). Dunnett’s post hoc test, with the C as reference, revealed that all nanoparticle treatments (N1, N2, N3) produced significantly higher fresh biomass compared to the control (*p* < 0.001 for N1 and N3; *p* = 0.001 for N2). In contrast, SC did not differ significantly from the control (*p* = 0.138). Bonferroni multiple comparisons confirmed these trends, with the largest positive effect observed for N3, which increased fresh biomass by 0.56 g relative to the control (Figure 4 and Figure 5, Table S2).

### 3.3. Effects of Silver Nanoparticles on Proline, Polyphenols, and Assimilatory Pigments Content in Wheat Leaves

#### 3.3.1. Proline Content

**Figure 6:**
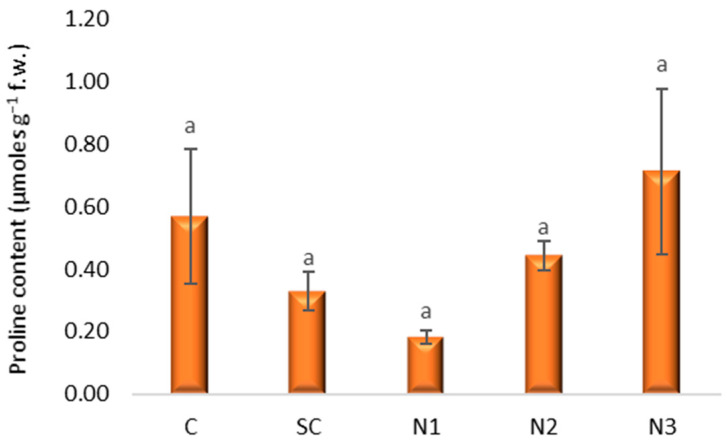
The influence of the AgNPs (20 nm, 0.02 mg/mL dispersed in a 2 mM sodium citrate solution, λmax: 405 nm) on proline content in *T. aestivum* leaves, 21 days after exposure; (a: the interpretation of the significance of the differences by means of the Duncan test, *N* = 15, df = 4, *p* < 0.05).

Proline content ranged from 0.81 µmoles g^−1^ f.w. in variant N1 to 0.71 µmoles g^−1^ f.w. in N3 (Figure 6). The 95% CIs are wide and include both decreases and increases relative to C, indicating imprecise estimates with this sample size (Table S3). A slight, non-significant inhibition of proline content was noted in the treatments exposed to higher AgNP concentrations (N1 and N2), whereas the highest dilution (N3) resulted in an insignificant stimulation after multiplicity adjustment (all adjusted *p* ≥ 30).

#### 3.3.2. Total Phenols Content

**Figure 7:**
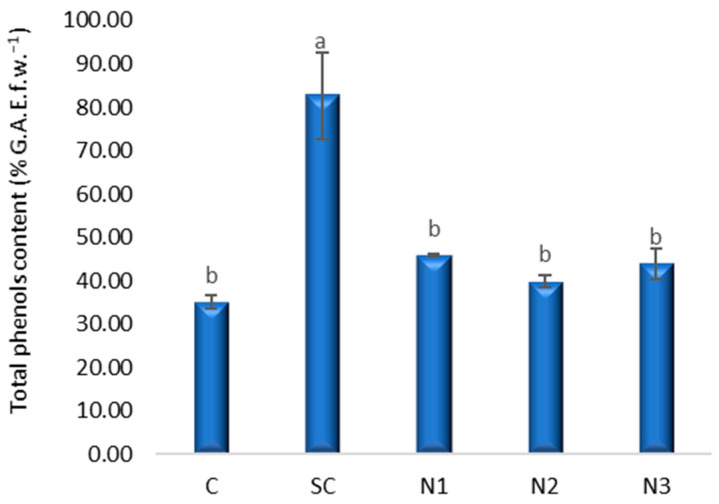
The influence of the AgNPs (20 nm, 0.02 mg/mL dispersed in a 2 mM sodium citrate solution, λmax: 405 nm) on polyphenols content in *T. aestivum* leaves, 21 days after exposure; (a, b: the interpretation of the significance of the differences by means of the Duncan test, *N* = 15, df = 4, *p* < 0.05).

The highest total phenol content was recorded in the solvent control at 80.26% G.A.E., f.w.^−1^ (Figure 7), with a large, precise increase in total phenols (adjusted *p* < 0.001; CI excludes zero by a wide margin; *g* ≈ 5.7). Although variants treated with AgNPs exhibited an increasing trend in total phenol content relative to the control, N1, N2, and N3 did not differ from control (adjusted *p* ≥ 0.37), and their CIs include zero. Pairwise Bonferroni results corroborate that SC exceeds each of N1–N3 (Table S3).

#### 3.3.3. Assimilatory Pigments Content

**Figure 8:**
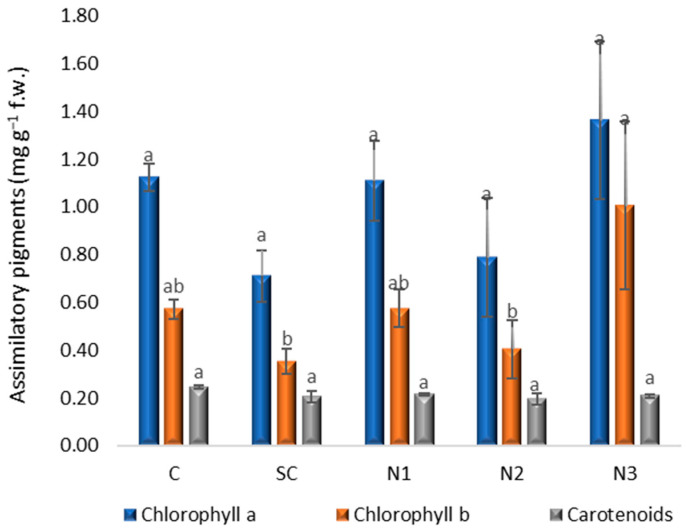
The influence of the AgNPs (20 nm, 0.02 mg/mL dispersed in a 2 mM sodium citrate solution, λmax: 405 nm) on assimilatory pigments content in *T. aestivum* leaves, 21 days after exposure; (a, b: the interpretation of the significance of the differences by means of the Duncan test, *N* = 15, df = 4, *p* < 0.05).

Assimilatory pigments serve as important biomarkers of plant physiological status, as increased chlorophyll synthesis is indicative of enhanced biomass production [27]. The highest concentrations of chlorophyll *a* and *b* were observed in the N3 variant, with values of 1.37 and 1.01 mg g^−1^ f.w., respectively (Figure 8). The one-way ANOVA did not reveal significant differences in chlorophyll *a* content (*F*(4, 10) = 1.67, *p* = 0.232) and chlorophyll *b* content, respectively (*F*(4, 10) = 2.198, *p* = 0.142). among the experimental variants. Post hoc Bonferroni tests confirmed the absence of significant pairwise differences between treatments. The highest mean difference in chlorophyll *a* content was observed between control (C) and SC (*p* > 0.46 in all comparisons), but it did not reach statistical significance. The Dunnett test comparing treatments with C also confirmed that none of the treatments diverged significantly from the control. Carotenoid content showed no significant variation among the treatments considering ANOVA analysis (*F*_4_,_10_ = 1.405, *p* = 0.301), Bonferroni post hoc comparisons and Dunnett’s test (Table S4). The lowest values for chlorophyll a (0.71 mg g^−1^ f.w.) and chlorophyll b (0.35 mg g^−1^ f.w.) were recorded in the solvent control, while the lowest carotenoid content (0.19 mg g^−1^ f.w.) was observed in variant N2 without reaching statistical significance (Mean difference = −0.05107, *p* = 0.145, 95% CI [−0.1173, 0.0125]).

#### 3.3.4. *Allium cepa* L. Root Cells Viability

**Figure 9:**
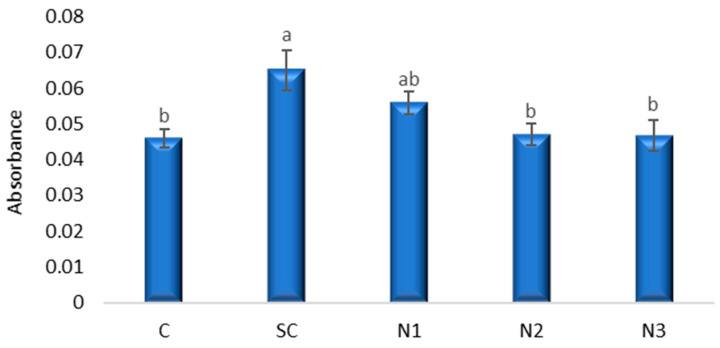
The influence of the AgNPs (20 nm, 0.02 mg/mL dispersed in a 2 mM sodium citrate solution, λmax: 405 nm) on cell viability in *A. cepa* root apex; (a, b: the interpretation of the significance of the differences by means of the Duncan test, *N* = 36, df = 35, *p* < 0.05).

In the current research the highest value of absorbance (0.065), which indicated that quantity of Evans blue absorbed by root meristematic cells, was recorded at the variant with SC (Figure 9). This value was significantly greater compared to those of control (C), N2 and N3 variants. Regarding the variants with AgNPs, the values determined at N2 and N3 are similar with that in control (C), while at N1 the number of death cells slightly increased as a consequence of the exposure to a higher concentration of NPs.

Bonferroni-adjusted multiple comparisons (Table S5) did not reveal statistically significant pairwise differences (*p* > 0.05), although near-significant trends of cell viability were observed between C and SC (mean difference = −0.019, *p* = 0.065, 95% CI [−0.0389, 0.0009]), and between SC and N2 (mean difference = 0.018, *p* = 0.089, 95% CI [0.019, 0.0379]).

Dunnett’s test, using C as the reference control, identified a significant increase in Evans blue uptake in the SC compared to C (mean difference = 0.019, *p* = 0.021, 95% CI [0.0029, 0.0351]). No significant differences were observed between C and AgNP treatments N1–N3 (*p* > 0.05).

### 3.4. The Effects Induced by AgNPs on the Mitotic Index, Distribution of Mitotic Phases and Genetic Material in Allium cepa L.

#### 3.4.1. Mitotic Index Variation and Distribution of Mitotic Phases

**Figure 10:**
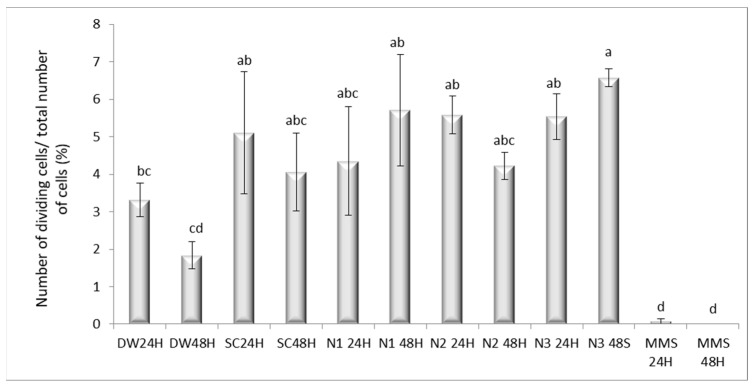
The influence of the AgNPs (20 nm, 0.02 mg/mL dispersed in a 2 mM sodium citrate solution, λmax: 405 nm) on the mitotic index in root meristematic cells of *A. cepa* (a–d: the interpretation of the significance of the differences by means of the Duncan test, *N* = 36, df = 35, *p* < 0.05).

The MI showed distinct variations across treatments and exposure times. The negative control (DW) exhibited a time-dependent decrease in mitotic activity, with MI dropping from 3.32% at 24 h to 1.84% at 48 h. Solvent control produced relatively high MI values: 5.11% at 24 h and 4.07% at 48 h (Figure 10). Complete mitotic inhibition was observed after exposure to MMS48H for which a mean difference of 5.107 (SE = 1.238), *p* = 0.029, 95% CI [0.268, 9.813] was calculated in Bonferroni-adjusted pairwise comparisons, while MMS24H also showed a negligible index (0.07%), the results being significantly lower compared to all other experimental variants. SC24H and SC48H did not induce dramatic decreases in the MI, their values being close to the negative control. However, SC induced a non-significant decrease in MI in a time-dependent manner.

The Bonferroni test showed limited significant pairwise differences due to the adjusted alpha level. Significant findings (*p* < 0.05) are summarized in Table S6. For most pairwise comparisons, differences between negative controls (DW24H, DW48H), solvent controls (SC24H, SC48H), and nanoparticle treatments (N1, N2, N3 at 24 h and 48 h) were not statistically significant (*p* > 0.05). In contrast, the positive control MMS (24 h and 48 h) displayed significantly lower MI values compared with several experimental groups. Specifically, MMS24H differed significantly from SC24H (*p* = 0.029), CI [−9.8129, −0.2683], N1 48H (*p* = 0.008), CI [−10.4167, −0.8721], N2 24H (*p* = 0.011), CI [−10.2968, −0.7522], N3 24H (*p* = 0.012), CI [−10.2474, −0.7028] and N3 48H (*p* = 0.001), CI [−11.2847, −1.7401]. Similarly, MMS48H showed significant differences versus SC24H (*p* = 0.025), N1 48H (*p* = 0.007), N2 24H (*p* = 0.009), N3 24H (*p* = 0.010), and N3 48H (*p* = 0.001). These findings confirm the strong mitoinhibitory effect of MMS compared to both controls and AgNP treatments.

When MMS48H was used as the comparison baseline, nearly all groups, including solvent controls and all AgNP treatments, displayed significantly higher MI values (*p* < 0.05) based on Dunnett’s test. For instance, SC24H (5.11, *p* = 0.003), SC48H (4.07, *p* = 0.025), N1 48H (5.71, *p* = 0.001), N2 24H (5.59, *p* = 0.001), and N3 48H (6.58, *p* < 0.001) all had significantly elevated mitotic indices relative to MMS48H. The only non-significant contrast was MMS24H vs. MMS48H (*p* = 1.000), confirming that both exposure times produced a comparable level of mitotic inhibition.

The most pronounced differences relative to MMS48H were observed in N3 48H (6.58, *p* < 0.001), N1 48H (5.71, *p* = 0.001), and N2 24H (5.59, *p* = 0.001), indicating that AgNP exposure did not reduce mitotic activity to the same extent as the positive control. Solvent controls also showed significantly higher MI compared to MMS, supporting the absence of cytogenotoxic effects from citrate alone.

**Figure 11:**
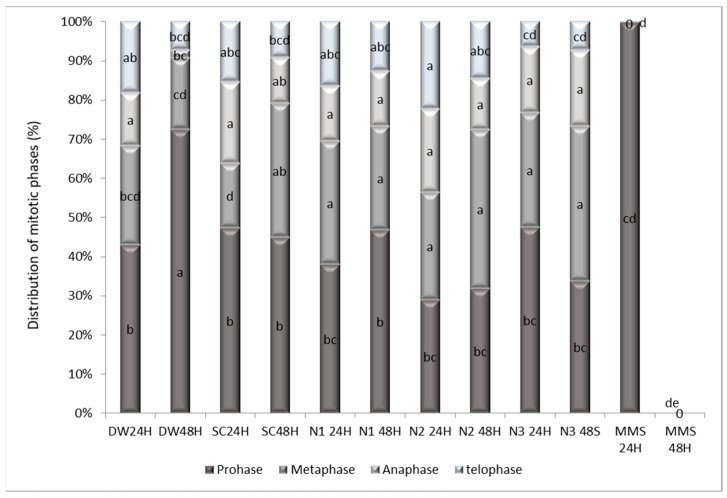
The influence of the AgNPs (20 nm, 0.02 mg/mL dispersed in a 2 mM sodium citrate solution, λmax: 405 nm) on the distribution of the mitosis phases in the meristematic root cells of *A. cepa* L. (a–e: interpretation of the significance of the differences, by means of the Duncan test, *N* = 36, df = 35, *p* < 0.05).

The distribution of cells in mitotic phases revealed significant changes under different treatments. The Bonferroni test indicated that the MMS 48H group exhibited a significantly higher prophase index than multiple groups, including AD24H (−42.86, *p* = 0.026), SC24H (−47.28, *p* = 0.009), SC48H (−44.82, *p* = 0.016), and N148H (−46.68, *p* = 0.01). Dunnett’s test (versus MMS 48 h as control) confirmed these findings. All groups, with the exception of MMS 24 h, showed significantly higher prophase indices compared to MMS 48 h (*p* < 0.05), including nanoparticle treatments (Figure 11, Table S7).

It is important to highlight those negative controls (DW and SC, both 24 h and 48 h) did not differ significantly from each other (*p* > 0.05), maintaining prophase index values similar to the negative baseline. Importantly, both controls differed significantly from MMS 48 h (*p* = 0.009–0.016, Bonferroni), validating their use as negative references.

Dunnett’s test showed the metaphase index of MMS 48H was significantly greater than all other treatment groups (all *p* < 0.05), including SC48H (34.28, *p* < 0.001), N1 24H (31.47, *p* < 0.001), and N2 48H (40.58, *p* < 0.001). This indicates that while other treatments may affect mitosis, none produced a metaphase arrest as severe as the positive control (Table S8).

Consistent with a blockade prior to anaphase onset, Dunnett’s test (Table S9) revealed that the anaphase index of the MMS 48H group was significantly lower than that of several groups, including SC24H (20.81, *p* = 0.002), N2 24H (21.34, *p* = 0.001), and N3 48S (17.22, *p* = 0.011). Conversely, for the telophase index, groups such as N2 24H (22.36, *p* = 0.005) and N2 48H (14.64, *p* = 0.037) scored significantly lower than the MMS 48H control, suggesting a more comprehensive arrest preventing exit from mitosis (Table S10).

#### 3.4.2. Chromosomal Aberrations Frequency

**Figure 12:**
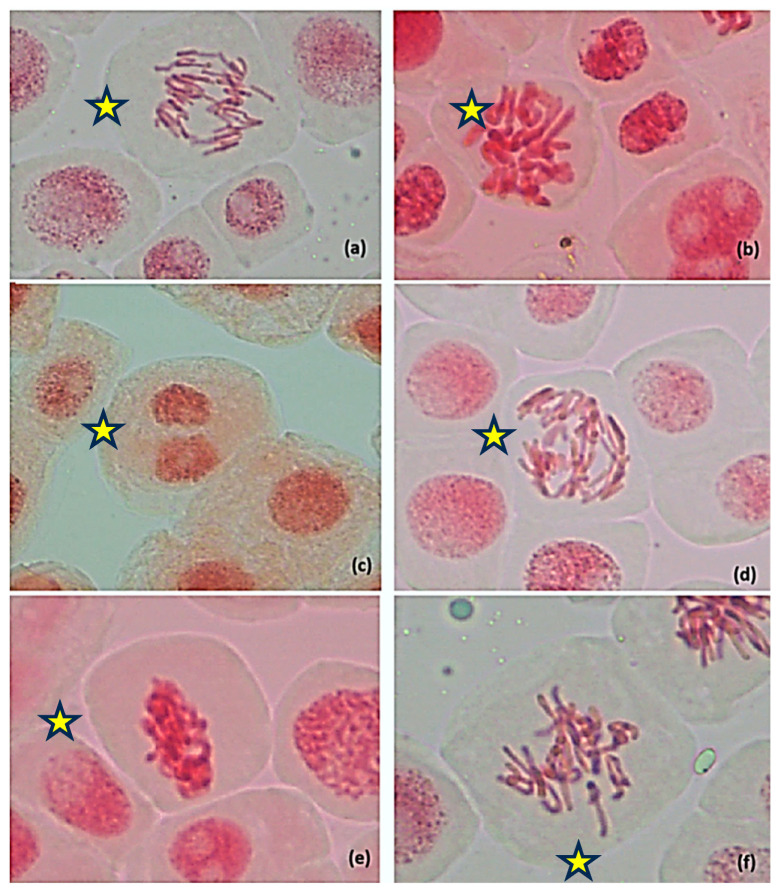
Chromosomal aberrations identified in root meristematic cells of *A. cepa* that underwent treatment with silver nanoparticles (20 nm, 0.02 mg/mL dispersed in a 2 mM sodium citrate solution, λmax: 405 nm); (**a**) Anaphase bridges, N2 24H; (**b**) C-mitosis, N1 24H; (**c**) binucleate cell, N1 48H; (**d**) multipolar anaphase anaphases showing chromosomes bridges—N2 24H; (**e**) Sticky chromosomes, N3 48S; (**f**) lagging chromosomes, N1 48H; 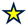
—cell with chromosomal aberrations, 400×. The original images of the chromosomal aberrations are provided in the Supplementary Material (Figures S3–S8).

AgNPs exposure induced various cytogenetic anomalies, including anaphase bridges, C-mitoses and sticky chromosomes, lagging chromosomes and multipolar anaphases as the most prominent ones (Figure 12).

The total frequency of aberrant cells varied substantially. The highest total aberration frequency was observed in SC48H (18.26%) and N2 24H (12.16%), while DW24H recorded only 2.74%. Anaphase bridges were the most frequent anomaly, with peak incidence in N2 24H (46.52%), followed by N3 48H (17.19%) and N1 48H (11.43%).

SC24H shows moderate levels of chromosomal aberrations, particularly lagging chromosomes (3.74 ± 3.7) and total aberrations (10.34 ± 3.25). At 48H, SC shows slightly increased total aberrations (18.26 ± 7.66), with C-mitosis (0.55 ± 0.35) and sticky chromosomes (0.17 ± 0.17) appearing, while lagging chromosomes decrease to zero.

Multipolar anaphases were detected only in N2 24H (5.59%); sticky chromosomes were noted in SC48H (0.17%), N1 24H (0.06%), and N3 48H (0.10%), while C-mitoses occurred with significant higher frequency in SC48H (0.55%), by means of the Duncan test, *p* < 0,05, and N1 48H (0.06%). Micronucleated and binucleated cells were rare, present in small percentages mainly in citrate controls and early AgNP treatments (Table 2). MMS-treated cells (24 h and 48 h) exhibited no mitotic activity and no detectable chromosomal aberrations, confirming their cytotoxic arrest.

Correlation analysis confirmed a significant relationship between mitotic inhibition and the induction of chromosomal aberrations. MI was significantly correlated with the aberration index (r = 0.337, *p* = 0.045), as well as with specific genotoxic endpoints such as anaphase bridges (r = 0.465, *p* = 0.004), lagging chromosomes (r = 0.343, *p* = 0.041), and stray chromosomes (r = 0.442, *p* = 0.007). Notably, the strongest correlation was observed with C-mitoses (r = 0.801, *p* < 0.001), consistent with a spindle poison-like effect (Table 3). These results support the existence of a quantitative dose–response relationship in which decreased mitotic activity is accompanied by increased chromosomal damage in *A. cepa* root meristems exposed to AgNPs.

## 4. Discussion

### 4.1. The Effects Induced by AgNPs on Axial Organ Growth and Seedling Weight of Triticum aestivum L.

The results obtained indicate a significant stimulatory effect of the treatment performed with the solution of AgNPs with a size of 20 nm, at a dilution of 10^−3^ (N3 variant −0.02 µg mL^−1^), compared to the control variants (C), in the two tested species, *T. aestivum* and *A. cepa* (Table 4).

The data demonstrate that AgNP treatments can differentially influence plants growth, with roots and stems responding variably. The significant increases in root length observed for N1 and N3 suggest that certain NP formulations can enhance belowground growth. In contrast, stem length was more responsive to N3, indicating that this experimental variant may promote coordinated growth of both organs, although Bonferroni comparisons suggest variability among replicates.

While SC treatment alone did not significantly alter either fresh or dry biomass compared with C, all AgNP treatments consistently stimulated biomass accumulation. The strongest effect was observed in the N3 variant, which produced increases of approximately 56% in wet biomass and 11% in dry biomass compared with C. Additionally, this variant exhibited the lowest percentage of chromosomal aberrations. Parameters such as cell viability, polyphenols, proline, and assimilatory pigments showed no significant changes compared to the control. Such responses may involve enhanced nutrient uptake, stimulation of phytohormone signaling or modulation of redox balance. Conversely, the lack of significant biomass changes in the SC group indicates that sodium citrate alone does not drive these effects, supporting the interpretation that NPs, rather than the stabilizing agent, are responsible for the observed biomass stimulation.

These findings are consistent with earlier reports that AgNPs at appropriate concentrations promote root and shoot growth through enhanced metabolic activity. The differences between wet and dry biomass further suggest that NPs may influence both water uptake and dry matter accumulation, with N3 showing the strongest capacity to improve overall biomass allocation.

For instance, AgNPs are known to promote plant growth, particularly during early germination stages [17], by enhancing the activity of growth hormones such as cytokinins, gibberellins, and auxins [31]. This hormonal modulation influences cell division and elongation processes [32,33], as supported by our results. Furthermore, the stimulatory effects may also be attributed to increased water absorption induced by lower AgNP concentrations [34]. Levard et al. [35] reported a slight, though statistically insignificant, increase in plant elongation at sub-toxic AgNP concentrations. Accordingly, the N3 variant in our experiment likely represents a sub-toxic concentration.

Previous studies indicate that citrate-coated AgNPs at 3 mg kg^−1^ (size 10–12 nm) do not negatively affect wheat growth [36]. Moreover, spherical AgNPs averaging 10 nm in size, within concentrations ranging from 0.06 to 0.5 mg L^−1^, have been proposed as an eco-friendly fertilizer alternative for wheat [37]. Additionally, AgNPs of similar size (10 nm) at 50–75 mg L^−1^ concentrations have demonstrated protective effects against heat stress in wheat plants [38].

The AgNPs using *T. aestivum* and *Zea mays* L. at 20 μg mL^−1^ increased growth, chlorophyll, and protein content in wheat and corn, consistent with our findings [14]. Similarly, a concentration of 25 mg L^−1^ of synthesized AgNPs (spherical shape, particle size 20–35 nm) significantly enhanced seed germination and early seedling growth of wheat compared to control on both the 4th and 8th days [39]. According to Kim et al. [40], *T. aestivum* roots exposed to up to 30 ppm AgNPs were slightly longer than controls.

The findings of our study underline the importance of assessing both root and shoot components when evaluating plant responses to NPs treatments. They also suggest that specific NP formulations, such as N3, may offer agronomic benefits by enhancing overall biomass production, although the variable stem response indicates that careful dose optimization and further mechanistic studies are needed to fully understand the underlying physiological and molecular processes.

### 4.2. Biochemical Responses of Triticum aestivum L. Seedling Leaves to AgNP Exposure

Our results are similar to those obtained by Nurlaelah et al. [17] who observed that biogenic AgNPs did not induce significant oxidative stress in soybean plants because there was no significant difference in proline content between control and AgNP treatment. Proline is an antioxidant that alleviates the adverse effects of reactive oxygen species (ROS) and its accumulation is important for adaptive responses [5] because its generation is essential to restore normal stress levels [41]. Atia and Oraibi [42] also observed the highest concentration of proline (23.8) of wheat cultivars Ibaa-99 in the variant with the smallest concentration of AgNPs (1 mg mL^−1^). Meanwhile, a decrease in proline content was observed for the wheat cultivar AlRasheed: significant at concentrations of 1 and 1.5 mg mL^−1^ of AgNPs and nonsignificant at 2 mg mL^−1^ compared with the control. Testing higher concentrations of AgNPs resulted in modifications of the proline content. There was an exponential increase in the proline level as AgNP concentration increased. Generally, high concentrations of AgNPs (40 and 50 mg L^−1^) resulted in high proline levels [5].

SC produced a robust elevation in phenolic content versus control and versus each of N1–N3. The Dunnett CI (+27.8 to +67.2) and very large standardized contrast (*g* ≈ 5.7) indicate a clear, practically important effect under these conditions. While SC was shown to reduce the biosynthesis of flavonoids and carotenoids [43], other studies revealed that SC increased the accumulation of phenolic compounds, enhanced overall antioxidant capacity, and inhibited key enzyme activities, thereby exerting a dual modulatory role on plant secondary metabolism [44]. The presence of AgNPs in the medium induced ROS production and, dependent on the dose used, increased total phenolic content [45]. High concentrations of AgNPs induced an increase in phenolic compounds content, but the mechanism of action has not yet been determined [15]. Jadczak et al. [16] reported that the polyphenol content was higher than in that in the control when the plants grew on media with 10–50 mg dm^−3^ AgNPs, but the lavender cultivated on the lowest AgNP concentrations (1 and 2 mg dm^−3^) was characterized by significantly lower polyphenol contents. The concentration of five phenolic compounds in *Arabidopsis thaliana* was elevated after nanoparticle treatment, indicating that the secondary metabolite profile changed after silver nanoparticle treatment [46]. The results obtained in the present research regarding the content of proline and polyphenols indicate low oxidative stress as a result of exposure to AgNPs, which determined a slight (non-significant) stimulation of polyphenol production compared to the control variant (M).

Recent research indicates changes in the content of assimilatory pigments as a result of plant exposure to different doses of AgNPs. Our results indicate that the low concentrations of AgNPs to which wheat caryopses were exposed (2–0.02 µg mL^−1^) caused insignificant changes in the content of assimilatory pigments, after a period of 21 days from exposure. However, chlorophyll *b* content under N3 treatment and carotenoid content under N2 treatment, although not significant, showed a potential stimulation and a slight reduction, respectively, which could indicate a treatment-specific trend toward increasing or diminished pigment accumulation. These patterns, although statistically inconclusive, may hint at subtle physiological responses that were not captured due to the limited replication (df = 10 within groups). Future studies with expanded sample sizes, or under stress conditions that challenge pigment stability, may clarify whether these trends represent biologically relevant effects or are random fluctuations. In practical terms, the lack of significant differences emphasizes the stability of photosynthetic pigments under the tested conditions, supporting the idea that the treatments did not impair primary photosynthetic machinery, especially in short-term exposure.

Various studies have highlighted the fact that the results obtained are dependent on the species, the ontogenetic moment investigated, the dose applied, and the additional stress to which the plant is exposed. Nurlaelah et al. [17] observed that the seedling of soybean exposed to AgNPs showed an increase in photosynthetic pigment contents. A concentration of 100 ppm AgNPs had a significant effect on the lily (*cultivar Little John*) leaf content of photosynthetic pigments determining the greatest amounts of chlorophyll *a*, chlorophyll *b*, chlorophyll *a* + *b*, and carotenoids [47].

Biba et al. [48] investigated the effects of 25, 50, 75, 100 and 150 µM AgNPs on early growth of tobacco (*Nicotiana tabacum* L.) and on photosynthetic performance and pigment content. They observed that root growth was significantly enhanced after exposure to lower AgNP concentrations (25 and 50 µM). Regarding the content of pigments, the highest concentration of total chlorophylls was measured in the variant with 50 μM AgNP concentration, and much more, a significant increase in total chlorophyll was obtained at almost all tested concentrations of AgNPs.

The application of AgNPs (0.025 g L^−1^) promoted shoot formation, improved chlorophyll *a*/*b*, and total/carotenoid ratios, as well as better levels of proline biosynthesis in response to stress (AlCl_3_) [49].

Shaikhaldein et al. [5] considered that the reason for the increased chlorophyll content of *Maerua oblongifolia* (Forsk.) A. Rich. plants after 20 mg L^−1^ AgNP exposure is the increase in nitrogen, magnesium, and iron concentrations, elements associated with chlorophyll biosynthesis. The much lower concentrations of AgNPs tested by us produced only a slight (insignificant) stimulation of chlorophyll *a* and *b* content.

### 4.3. Assessment of Cell Viability Alterations in Allium cepa L. Following AgNPs Exposure

Cell death can be induced by various factors (abiotic stresses including drought and metals), and measuring cell viability using Evans staining is an accepted technique [50]. Evans blue only stains dead cells because in living cells it is enzymatically reduced to a colorless form [51]. The Evans blue assay revealed that membrane permeability was significantly affected by the SC, which differed from the untreated C. This suggests that the solvent used for NP suspension may exert measurable stress on cellular membranes, independent of NP exposure. Such effects are consistent with earlier studies reporting solvent-mediated cytotoxicity in various human cell lines [52,53].

Although the ANOVA confirmed significant group-level differences, most Bonferroni pairwise comparisons failed to reach significance, reflecting the conservative nature of this correction. Nonetheless, the consistent near-significant effects observed for SC relative to both C and N2 suggest that the solvent may have a modest but biologically relevant impact on membrane integrity.

In contrast, the NP treatments (N1–N3) did not significantly increase Evans blue uptake compared with C, indicating that under the tested conditions, AgNPs did not induce marked plasma membrane damage. This finding could reflect either the use of concentrations below the threshold for acute cytotoxicity or a more subtle mode of action affecting intracellular processes rather than direct membrane disruption.

The AgNP effects on cell division can be investigated in the root apex because there are undifferentiated cells [54]. After studying the AgNP impacts on root development in *A. cepa* [55], Kumari et al. [56] observed a reduction in mitochondrial activity and also suggested that root tip cells can readily internalize AgNPs. AgNPs need to penetrate the cell wall and plasma membranes of epidermal layer of roots so that small-sized AgNPs can pass through the pores [57].

Kannaujia et al. [58] studied the cell viability of radicles from two wheat varieties (HD-2967 and DBW-17) after their exposure to AgNPs. The treatment with 10 mg L^−1^ AgNPs effectively promoted the early growth of wheat seedlings by decreasing ROS toxicity. Root cell viability assessed by Evans Blue staining reached the maximum in wheat radicles from the HD-2967 variety treated with AgNPs, while in the DBW-17 variety, the maximum root cell viability was observed in the control and was close to that of the AgNP-treated variant. The major deteriorations of cells in roots of *T. aestivum* seedlings treated with the smallest AgNPs (10 and 20 nm) were observed by Lahuta et al. [7] in the apical meristem region and elongation zone. A slight insignificant deterioration in cell viability was also observed in the current experiment, following exposure of *A. cepa* root meristem to low doses of AgNPs (2, 0.2, and 0.02 µg mL^−1^, respectively), cell viability being affected in direct proportion to the concentration of AgNPs.

### 4.4. Cytogenetic and Mitotic Responses of A. cepa Root Meristems to AgNPs Exposure

The treatments with AgNPs (N1, N2, N3) showed intermediate values, positioned between the positive control and the negative controls. Compared to MMS, all these treatments exhibited significant differences (*p* < 0.05, Dunnett), indicating a much lower inhibition of mitosis. Conversely, strong inhibition of cell division observed under genotoxic stress supports the utility of positive controls such as MMS in validating assay sensitivity. The complete arrest of mitosis following MMS exposure underscores its capacity to disrupt DNA integrity through alkylation, leading to mitotic block [59,60].

However, among all treatments, N1, N2, and N3 did not display significant differences (*p* > 0.05, Bonferroni), suggesting a similar effect within the tested concentration range. However, the test compounds N2 and N3 display a statistically similar profile to MMS in their effect on the prophase index. The lack of significant difference from the positive control (Dunnett’s test, *p* > 0.05) suggests that these compounds may share a genotoxic mechanism of action, potentially causing DNA damage that triggers the G2/M checkpoint. In contrast, compounds DW and N1 appear to be less potent, as their indices were consistently and significantly lower than the MMS control. Furthermore, the depression of the telophase index in the N2-treated groups implies an additional or stronger block late in mitosis, preventing cytokinetic completion, a phenomenon that warrants further mechanistic investigation.

The observed modulation of mitotic activity following exposure to AgNPs suggests a dose- and time-dependent impact on cellular proliferation processes, consistent with the dynamic responses reported in previous studies [61,62]. At lower concentrations and longer exposure durations, AgNPs appear to stimulate mitotic division in a non-significant stimulatory trend, a biphasic phenomenon whereby mild stressors trigger adaptive cellular activity. Such mitogenic stimulation has been described in plant meristem systems exposed to low levels of various NPs [63,64,65].

The observed stimulatory effect at the lowest AgNP concentration is consistent with previous reports on nanoparticle-plant interactions. This stimulatory effect may be mediated by enhanced biosynthesis and signaling of key phytohormones, including abscisic acid (ABA) and auxins [66], which are known to regulate root growth and development. Additionally, this indirect correlated response appears to be organ-specific, with roots exhibiting a greater stimulation than shoots, likely reflecting a strategic allocation of resources to organs experiencing mild stress in contact with AgNPs [4,67].

In aging root tips, a physiological reduction in mitotic activity was noted, consistent with the decline in meristematic vigor over time. This highlights the importance of temporal factors when evaluating MI in untreated controls. The physiological decline in mitotic activity observed in aging root tips reflects a temporal gradient along the root axis, wherein reduced cell production in the meristem, particularly under stress conditions, leads to fewer and smaller cells entering the elongation zone, ultimately resulting in a shortened growth zone due to impaired cell division rather than altered elongation timing [68,69]. Türkoğlu [70] recorded a reduced mitotic division in roots of *A. cepa* treated with sodium citrate in concentrations ranging from 20 to 100 ppm for 5, 10 and 20 h. Meanwhile, in our study the lack of significant increase in MI associated with 2 mM sodium citrate treatment for 24 and 48 h confirms its suitability as a biologically inert dispersant under experimental conditions.

Together, these findings reinforce the dualistic nature of AgNPs in modulating plant cell division. At subtoxic levels, they may transiently enhance proliferation, potentially through mild oxidative or structural stress. However, at elevated doses or with prolonged exposure, their accumulation likely triggers cytotoxic pathways that impair mitotic progression. This nuanced response emphasizes the importance of precise concentration and exposure control in nanotoxicological assessments.

The distribution of mitotic phases reflects the integrity of cell cycle progression and the functionality of associated checkpoints [71]. Alterations in phase ratios across treatments suggest differential impacts on mitotic regulation. AgNPs exposure appeared to partially preserve phase distribution under certain conditions, though specific treatment regimens were associated with metaphase accumulation. This may point to disruptions in spindle formation or function, with a possible involvement of the spindle assembly checkpoint (SAC). SAC activation is typically triggered by improper kinetochore-microtubule attachments or spindle disorganization, hallmarks of microtubule-targeting agents. These findings are consistent with prior studies demonstrating that NPs can exert both mitostimulatory and mitodepressive effects depending on dosage, exposure time, and nanoparticle characteristics such as size and surface chemistry [55,72].

From a practical standpoint, the biphasic effects of AgNPs, mitotic stimulation at low concentrations and cytogenetic disturbances at higher concentrations, respectively, underscore the necessity of carefully defining safe exposure thresholds in agricultural applications. Establishing such thresholds would require systematic dose–response studies under agronomically relevant conditions, accounting for both short- and long-term effects on plant growth, productivity, and genomic stability. This approach would enable harnessing potential beneficial effects at low doses while minimizing genotoxic risks at higher exposures.

Mechanistically, the mitotic spindle plays a central role in chromosome segregation, and its disturbance often results in aneuploidy, a condition frequently associated with tumorigenesis [73]. In plants, although centrosome-like structures are absent, the spindle assembly checkpoint is functionally conserved. However, its regulatory architecture differs markedly from that of animals and yeast. In *Arabidopsis*, SAC activation is transient, lasting less than two hours, after which mitotic arrest is reset. This feature may facilitate genome duplication and promote polyploidization, offering potential evolutionary advantages under environmental stress [74,75].

Overall, treatment-specific phase shifts suggest that AgNPs can interfere with the fidelity of mitotic spindle formation and checkpoint regulation, thus altering cell cycle dynamics. These effects underscore the importance of dose and exposure time in modulating nanoparticle toxicity and support the growing body of evidence linking AgNP exposure to both genotoxic and mitotic outcomes.

The exposure to AgNPs induced a broad spectrum of mitotic and cytogenetic anomalies in *A. cepa* root tips. The high incidence of anaphase bridges, bulky DNA structures, likely reflects telomere-to-telomere fusion involving dicentric chromosomes or sister-chromatid fusion, hallmark indicators of clastogenic activity. These structures can initiate the breakage-fusion-bridge (BFB) cycle, leading to chromosomal instability. If not properly resolved during anaphase or telophase, anaphase bridges can impede cytokinesis, induce tetraploidy, and cause DNA breakage, which may trigger further chromosomal rearrangements such as translocations [76,77,78].

Additionally, the frequent occurrence of C-mitoses and sticky chromosomes, particularly in the N1 24H and N2 24H groups, suggests that AgNPs interfere with spindle apparatus function and chromatin condensation, disrupting normal mitotic progression. It is important to note that the higher frequency of total aberrations observed in SC24H compared with SC48H suggests a time-dependent effect of the solvent itself. Consistent with this, Türkoğlu [70] reported that sodium citrate reduced the mitotic index of *Allium cepa* root meristems in a concentration- and time-dependent manner, while also inducing chromosomal aberrations such as bridges, C-mitosis, lagging chromosomes, and micronuclei.

Notably, lagging chromosomes and multipolar anaphases were also observed, indicative of errors in kinetochore-microtubule interactions and spindle pole organization. Lagging chromosomes arise from mitotic errors such as abnormal microtubule–kinetochore attachments, centrosome amplification, defective spindle assembly checkpoints, and sister chromatid cohesion defects. A frequent cause is merotelic attachment, where a single kinetochore is attached to both spindle poles [79]. Lagging chromosomes that fail to reintegrate into daughter nuclei may be lost or form micronuclei, contributing to chromosomal instability [80].

These anomalies further support the hypothesis that AgNPs can act as aneugens by perturbing chromosome segregation fidelity. The total frequency of mitotic aberrations was significantly higher in the SC48H and N2 24H groups compared to the negative control, emphasizing the genotoxic potential of AgNPs, particularly under prolonged or higher-dose exposure conditions. Literature evidence from *A. cepa* assays suggests that some genotoxic effects, particularly spindle-related aberrations, can be partially reversible upon removal of the stressor, whereas structural chromosomal aberrations tend to persist [20,81]. Therefore, while our correlation data support a quantitative dose–response relationship in which decreased mitotic activity is accompanied by increased chromosomal damage in *A. cepa* root meristems exposed to AgNPs, further experiments incorporating a recovery phase would be required to determine the extent of reversibility for AgNP-induced cytogenetic damage.

Given the increasing interest in the use of AgNPs in agriculture as nano-pesticides and nano-fertilizers [33], our findings raise important considerations regarding their potential long-term effects on plant development and genomic stability. While our data do not indicate direct agronomic risks, they emphasize the need for further studies under field-relevant conditions, including chronic exposure scenarios and whole-plant assessments. Such investigations are critical to determine whether the cytogenetic effects observed in the *Allium* test translate into meaningful consequences for crop growth, yield, or ecosystem balance.

Moreover, the results highlight the dualistic concentration- and time-dependent behavior of AgNPs, which can induce both mitotic stimulation and inhibition. This biphasic response reflects the complex nature of nanoparticle–cell interactions and underscores the necessity for detailed dose–response analyses in toxicological and ecological evaluations. Finally, our study demonstrates the importance of monitoring both structural and numerical chromosomal abnormalities to provide a comprehensive assessment of the genotoxic potential of engineered nanomaterials.

## 5. Conclusions

The AgNPs (2.00, 0.2 and 0.02 µg mL^−1^) dispersed in a 2 mM sodium citrate solution, with dimensions of 20 nm, applied to *T. aestivum*, at the caryopsis stage, exhibited stimulatory effects on growth parameters, with the most pronounced effects observed at the lowest concentration (0.02 µg mL^−1^), which significantly enhanced both axial organ length and seedling biomass. While low AgNP concentrations mildly stimulated mitotic activity, higher doses caused notable chromosomal aberrations and altered phase ratios in the cell cycle. The non-enzymatic antioxidant response was minimally affected, showing only a slight, non-significant increase in polyphenol production. AgNPs exert concentration- and time-dependent cytogenotoxic effects on *A. cepa* root meristem cells. The findings underline the potential of AgNPs to interfere with cell cycle regulation and genomic stability, raising concerns about their environmental and biological impact. The *Allium* test proved effective for detecting nanoparticle-induced genotoxicity and can be recommended as a primary screening assay for nanoparticle safety evaluation.

## Figures and Tables

**Table 1:** Experimental treatment groups and corresponding AgNP concentrations.

Nr. Crt.	Variant Code	Content	Dilution
1	S	Negative control: distilled water	-
2	SC	Solvent control: Sodium citrate 2 mM	-
3	N1	AgNPs dispersed in sodium citrate 2 mM	10^−1^ (2 µg mL^−1^)
4	N2	AgNPs dispersed in sodium citrate 2 mM	10^−2^ (0.2 µg mL^−1^)
5	N3	AgNPs dispersed in sodium citrate 2 mM	10^−3^ (0.02 µg mL^−1^)

**Table 2:** Chromosomal aberrations observed in meristematic root cells of *A. cepa* exposed to silver nanoparticles (20 nm, 0.02 mg/mL dispersed in a 2 mM sodium citrate solution, λmax: 405 nm) (a–d: interpretation of the significance of the differences, by means of the Duncan test, *p* < 0.05, *N* = 36, df = 35).

Experimental Variant	Binucleated Cells	Lagging Chromosomes	C-Mitosis	Sticky Chromosomes	Anaphase Bridges	Multipolar Anaphases	Other Aberrations	Total Aberrations
DW24H	0.00	0.00	0.00	0.00	22.22 ± 14.7 ab	0.00	0.00	2.74 ± 1.71 cd
DW48H	0.00	0.00	0.00	0.00	0.00	0.00	0.00	0.00
SC24H	0.03 ± 0.0 a	3.74 ± 3.7 a	0.00	0.1 ± 0.06 a	22.22 ± 11.11 ab	0.00	1.85 ± 1.85 a	10.34 ± 3.25 abc
SC48H	0.00	0.00	0.55 ± 0.35 a	0.17 ± 0.17 a	8.33 ± 8.33 b	0.00	2.78 ± 2.78 a	18.26 ± 7.66 a
N1 24H	0.00	0.00	0.03 ± 0.03 b	0.06 ± 0.06 a	11.11 ± 7.35 b	0.00	0.00	4.96 ± 0.92 bd
N1 48H	0.04 ± 003 a	0.07 ± 0.07 a	0.06 ± 0.06 b	0.00	11.43 ± 5.95 b	0.00	6.06 ± 6.06 a	5.6 ± 1.87 bcd
N2 24H	0.00	0.00	0.07 ± 0.07 b	0.00	46.52 ± 7.66 a	5.59 ± 2.82 a	0.00	12.16 ± 3.05 ab
N2 48H	0.00	0.00	0.03 ± 0.03 b	0.00	16.66 ± 16.67 b	0.00	0.00	3.26 ± 2.18 bcd
N3 24H	0.00	0.00	0.00	0.00	14.99 ± 14.68 b	0.00	0.00	3.1 ± 0.57 bcd
N3 48S	0.00	0.00	0.00	0.1 ± 0.06 a	17.19 ± 5.19 b	0.00	1.67 ± 1.67 a	5.05 ± 0.41 bcd
MMS 24H	0.00	0.00	0.00	0.00	0.00	0.00	0.00	0.00
MMS 48H	0.00	0.00	0	0.00	0.00	0.00	0.00	0.00

**Table 3:** Correlation Analysis of Mitotic Activity and Chromosomal Aberrations in the *Allium* Test.

Experimental Variants	Mitotic Index	Prophase Index	Metaphase Index	Anaphase Index	Telophase Index	Binucleated Cells	Lagging Chromosomes	C-Mitosis	Stickies	Anaphase Bridges	Multipolar Anaphases	Other Aberrations	Total Aberrations
Mitotic index	1	0.314	0.531 **	0.661 **	0.377 **	0.21	0.442 **	0.038	0.29	0.465	0.222	0.122	0.337 **
Prophase index	0.314	1	0.21	0.056	0.173	0.228	0.217	0.269	−0.034	0.033	0.2	0.032	0.275
Metaphase index	0.531 **	0.21	1	0.502 **	0.397 *	−0.147	−0.042	0.144	0.164	0.317	0.058	0.077	0.275
Anaphase index	0.661 **	0.056	0.502 **	1	0.458 **	0.233	0.062	−0.168	0.273	0.59 **	0.206	0.205	0.324
Telophase index	0.377 *	0.173	0.397 *	0.458 **	1	−0.035	−0.032	0.093	0.29	0.297	0.296	−0.046	0.133
Binucleated cells	0.21	0.228	−0.147	0.233	-0.035	1	0.639 **	−0.071	−0.09	0.17	−0.059	0.073	0.195
Lagging chromosomes	0.442 **	0.217	−0.042	0.062	−0.032	0.639 **	1	−0.068	−0.105	0.137	−0.109	0.057	0.063
C-mitosis	0.038	0.269	0.144	−0.168	0.093	−0.071	−0.068	1	−0.104	−0.129	−0.035	0.433 **	0.801 **
Stickies	0.29	−0.034	0.164	0.273	0.29	−0.09	−0.105	−0.104	1	−0.156	−0.09	−0.012	0.188
Anaphase bridges	0.465 **	0.033	0.317	0.59 **	0.297	0.17	0.137	−0.129	−0.156	1	0.472 **	0.118	0.364 **
Multipolar anaphases	0.222	0.2	0.058	0.206	0.296	−0.059	−0.109	−0.035	−0.09	0.472 **	1	-0.073	0.258
Other aberrations	0.122	0.032	0.077	0.205	−0.046	0.073	0.057	0.433 **	−0.012	0.118	−0.073	1	0.391 *
Total aberrations	0.337 *	0.275	0.275	0.324	0.133	0.195	0.063	0.801 **	0.188	0.364 *	0.258	0.391 *	1

** Correlation is significant at the 0.01 level (2-tailed). * Correlation is significant at the 0.05 level (2-tailed).

**Table 4:** Synthesis of significant changes induced by silver nanoparticle (20 nm, dispersed in a 2 mM sodium citrate solution) in *T. aestivum* and *A. cepa*.

Evaluated Parameter	Experimental Variants									
	C		SC		N1 2 µg mL^−1^		N2 0.2 µg mL^−1^		N3 0.02 µg mL^−1^	
	Significant Changes									
	+	−	+	−	+	−	+	−	+	−
Root length					+				+	
Shoot length									+	
Fresh weight			+		+		+		+	
Dry weight					+		+		+	
Proline										
Polyphenols			+							
Assimilatory pigments										
Cell viability				−						
Mitotic index									+	
Total chromosomal aberrations										−

+ significantly increased, − significantly decreased.

## Data Availability

The original contributions presented in this study are included in the article/Supplementary Material. Further inquiries can be directed to the corresponding author.

## Supplementary Materials

The following supporting information can be downloaded at: https://www.mdpi.com/article/10.3390/jox15050147/s1, Figure S1: AgNPs dispersion and form analysis performed by BF-STEM; Figure S2: *Triticum aestivum* L. plants, 21 days after AgNPs exposure; Figure S3: Chromosomal aberrations identified in root meristematic cells of *A. cepa* that underwent treatment with silver nanoparticles (20 nm, 0.02 mg/mL dispersed in a 2 mM sodium citrate solution, λmax: 405 nm): aAnaphase bridges, N2 24H; Figure S4: Chromosomal aberrations identified in root meristematic cells of *A. cepa* that underwent treatment with silver nanoparticles (20 nm, 0.02 mg/mL dispersed in a 2 mM sodium citrate solution, λmax: 405 nm): C-mitosis, N1 24H; Figure S5: Chromosomal aberrations identified in root meristematic cells of *A. cepa* that underwent treatment with silver nanoparticles (20 nm, 0.02 mg/mL dispersed in a 2 mM sodium citrate solution, λmax: 405 nm): binucleate cell, N1 48H; Figure S6: Chromosomal aberrations identified in root meristematic cells of *A. cepa* that underwent treatment with silver nanoparticles (20 nm, 0.02 mg/mL dispersed in a 2 mM sodium citrate solution, λmax: 405 nm): multipolar anaphase anaphases showing chromosomes bridges—N2 24H; Figure S7: Chromosomal aberrations identified in root meristematic cells of *A. cepa* that underwent treatment with silver nanoparticles (20 nm, 0.02 mg/mL dispersed in a 2 mM sodium citrate solution, λmax: 405 nm): sticky chromosomes, N3 48S; Figure S8: Chromosomal aberrations identified in root meristematic cells of *A. cepa* that underwent treatment with silver nanoparticles (20 nm, 0.02 mg/mL dispersed in a 2 mM sodium citrate solution, λmax: 405 nm): lagging chromosomes, N1 48H; Table S1: Post-hoc pairwise comparisons of root length and stem length across treatment groups using Bonferroni and Dunnett’s tests; Table S2: Post-hoc pairwise comparisons of fresh weight and dry weight across treatment groups using Bonferroni and Dunnett’s tests; Table S3: Post-hoc pairwise comparisons of proline and total phenols content across treatment groups using Bonferroni and Dunnett’s tests; Table S4: Post-hoc pairwise comparisons of assimilatory pigments content across treatment groups using Bonferroni and Dunnett’s tests; Table S5: Post-hoc pairwise comparisons of cell viability across treatment groups using Bonferroni and Dunnett’s tests; Table S6: Post-hoc pairwise comparisons of mitotic index across treatment groups using Bonferroni and Dunnett’s tests; Table S7: Post-hoc pairwise comparisons of prophase index across treatment groups using Bonferroni and Dunnett’s tests; Table S8: Post-hoc pairwise comparisons of metaphase index across treatment groups using Bonferroni and Dunnett’s tests; Table S9: Post-hoc pairwise comparisons of anaphase index across treatment groups using Bonferroni and Dunnett’s tests; Table S10: Post-hoc pairwise comparisons of telophase index across treatment groups using Bonferroni and Dunnett’s tests.

## Abbreviations

The following abbreviations are used in this manuscript:

AgNPs	silver nanoparticles
BFSTEM	bright field scanning transmission electron microscopy
d.w.	dry weight
f.w.	fresh weight
G.A.E.	gallic acid equivalents
MMS	methyl methane sulfonate
ROS	reactive oxygen species
SAC	spindle assembly checkpoint
SDS	sodium dodecyl sulfate
SE	standard deviation
