# Supplementary material for: Integrated Assessment of Silver Nanoparticles on Plant Growth and Cytogenotoxicity Using Triticum and Allium Bioassays
Source: J Xenobiot. 2025 Sep 12;15(5):147. doi: 10.3390/jox15050147 (PMC12452658; doi:10.3390/jox15050147)
Supplement: Supplementary file 1 [file jox-15-00147-s001.zip › jox-3808429-supplementary.pdf]

Journal of Xenobiotics

Supplementary Materials: Integrated Assessment of Silver Nanoparticles on Plant Growth and Cytogenotoxicity Using Triticum and Allium Bioassays

Simona Elena Pisculungeanu, Liliana Cristina Soare, Oana Alexandra Luțu, Alina Păunescu, Georgiana Cîrstea, Aurelian Denis Negrea, Codruța Mihaela Dobrescu and Nicoleta Anca Ionescu (Șuțan)

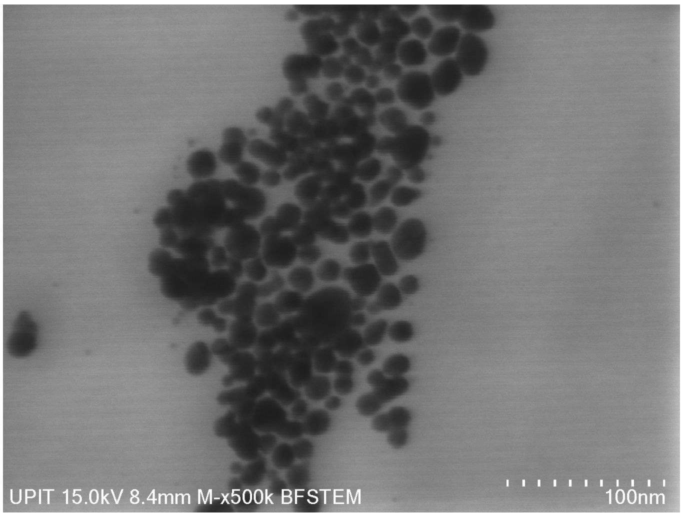

Figure S1. AgNPs dispersion and form analysis performed by BF-STEM.

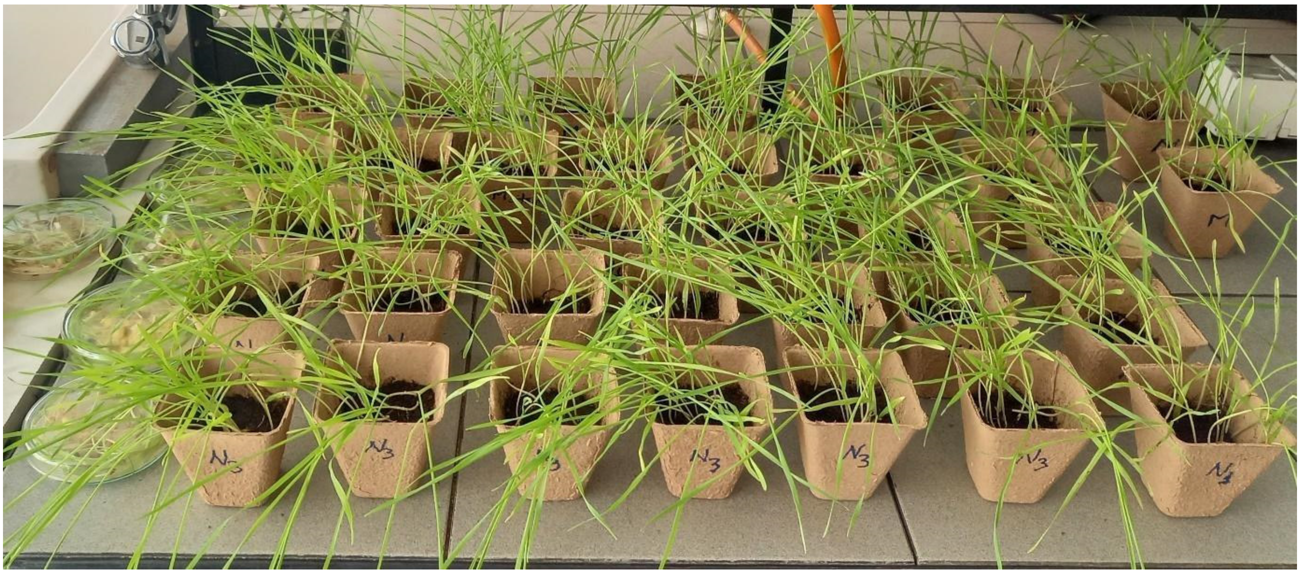

Figure S2. *Triticum aestivum* L. plants, 21 days after AgNPs exposure.

Table S1. Post-hoc pairwise comparisons of root length and stem length across treatment groups using Bonferroni and Dunnett's tests

Dependent Variable		(I) Variants	(J) Variants	Mean Difference (I-J)	Std. Error	Sig.	95% Confidence Interval	
							Lower Bound	Upper Bound
Roots	Bonferroni	C	SC	-20.66667	10.77600	.571	-51.3850	10.0517
			N1	-27.40000	10.77600	.120	-58.1183	3.3183
			N2	-14.76667	10.77600	1.000	-45.4850	15.9517
			N3	-35.90000*	10.77600	.011	-66.6183	-5.1817
		SC	C	20.66667	10.77600	.571	-10.0517	51.3850
			N1	-6.73333	10.77600	1.000	-37.4517	23.9850
			N2	5.90000	10.77600	1.000	-24.8183	36.6183
			N3	-15.23333	10.77600	1.000	-45.9517	15.4850
		N1	C	27.40000	10.77600	.120	-3.3183	58.1183
			SC	6.73333	10.77600	1.000	-23.9850	37.4517
			N2	12.63333	10.77600	1.000	-18.0850	43.3517
			N3	-8.50000	10.77600	1.000	-39.2183	22.2183
		N2	C	14.76667	10.77600	1.000	-15.9517	45.4850
			SC	-5.90000	10.77600	1.000	-36.6183	24.8183
			N1	-12.63333	10.77600	1.000	-43.3517	18.0850
			N3	-21.13333	10.77600	.518	-51.8517	9.5850
		N3	C	35.90000*	10.77600	.011	5.1817	66.6183
			SC	15.23333	10.77600	1.000	-15.4850	45.9517
			N1	8.50000	10.77600	1.000	-22.2183	39.2183
			N2	21.13333	10.77600	.518	-9.5850	51.8517
	Dunnett t (2-sided)[b]	SC	C	20.66667	10.77600	.175	-5.9391	47.2724
		N1	C	27.40000*	10.77600	.041	.7942	54.0058
		N2	C	14.76667	10.77600	.452	-11.8391	41.3724
		N3	C	35.90000*	10.77600	.004	9.2942	62.5058
Stem	Bonferroni	C	SC	-9.46667	8.01589	1.000	-32.3170	13.3836
			N1	-14.56667	8.01589	.712	-37.4170	8.2836
			N2	-6.16667	8.01589	1.000	-29.0170	16.6836
			N3	-24.70000*	8.01589	.025	-47.5503	-1.8497
		SC	C	9.46667	8.01589	1.000	-13.3836	32.3170
			N1	-5.10000	8.01589	1.000	-27.9503	17.7503
			N2	3.30000	8.01589	1.000	-19.5503	26.1503
			N3	-15.23333	8.01589	.594	-38.0836	7.6170
		N1	C	14.56667	8.01589	.712	-8.2836	37.4170
			SC	5.10000	8.01589	1.000	-17.7503	27.9503
			N2	8.40000	8.01589	1.000	-14.4503	31.2503
			N3	-10.13333	8.01589	1.000	-32.9836	12.7170
		N2	C	6.16667	8.01589	1.000	-16.6836	29.0170
			SC	-3.30000	8.01589	1.000	-26.1503	19.5503
			N1	-8.40000	8.01589	1.000	-31.2503	14.4503
			N3	-18.53333	8.01589	.222	-41.3836	4.3170
		N3	C	24.70000*	8.01589	.025	1.8497	47.5503
			SC	15.23333	8.01589	.594	-7.6170	38.0836
			N1	10.13333	8.01589	1.000	-12.7170	32.9836
			N2	18.53333	8.01589	.222	-4.3170	41.3836
	Dunnett t (2-sided)[b]	SC	C	9.46667	8.01589	.583	-10.3244	29.2578
		N1	C	14.56667	8.01589	.213	-5.2244	34.3578
		N2	C	6.16667	8.01589	.857	-13.6244	25.9578
		N3	C	24.70000*	8.01589	.009	4.9089	44.4911

*. The mean difference is significant at the 0.05 level.

b. Dunnett t-tests treat one group as a control, and compare all other groups against it.

Table S2. Post-hoc pairwise comparisons of fresh weight and dry weight across treatment groups using Bonferroni and Dunnett's tests

Dependent Variable		(I) Variants	(J) Variants	Mean Difference (I-J)	Std. Error	Sig.	95% Confidence Interval	
							Lower Bound	Upper Bound
Wet biomass	Bonferroni	C	SC	-.11000	.04867	.474	-.2843	.0643
			N1	-.38333*	.04867	.000	-.5576	-.2090
			N2	-.28000*	.04867	.002	-.4543	-.1057
			N3	-.55667*	.04867	.000	-.7310	-.3824
		SC	C	.11000	.04867	.474	-.0643	.2843
			N1	-.27333*	.04867	.002	-.4476	-.0990
			N2	-.17000	.04867	.058	-.3443	.0043
			N3	-.44667*	.04867	.000	-.6210	-.2724
		N1	C	.38333*	.04867	.000	.2090	.5576
			SC	.27333*	.04867	.002	.0990	.4476
			N2	.10333	.04867	.597	-.0710	.2776
			N3	-.17333	.04867	.052	-.3476	.0010
		N2	C	.28000*	.04867	.002	.1057	.4543
			SC	.17000	.04867	.058	-.0043	.3443
			N1	-.10333	.04867	.597	-.2776	.0710
			N3	-.27667*	.04867	.002	-.4510	-.1024
		N3	C	.55667*	.04867	.000	.3824	.7310
			SC	.44667*	.04867	.000	.2724	.6210
			N1	.17333	.04867	.052	-.0010	.3476
			N2	.27667*	.04867	.002	.1024	.4510
	Dunnett t (2-sided)[b]	SC	C	.11000	.04867	.138	-.0307	.2507
		N1	C	.38333*	.04867	.000	.2427	.5240
		N2	C	.28000*	.04867	.001	.1393	.4207
		N3	C	.55667*	.04867	.000	.4160	.6973
Dry biomass	Bonferroni	C	SC	-.04000	.03070	1.000	-.1499	.0699
			N1	-.11000*	.03070	.050	-.2199	-.0001
			N2	-.09333	.03070	.124	-.2033	.0166
			N3	-.11333*	.03070	.042	-.2233	-.0034
		SC	C	.04000	.03070	1.000	-.0699	.1499
			N1	-.07000	.03070	.458	-.1799	.0399
			N2	-.05333	.03070	1.000	-.1633	.0566
			N3	-.07333	.03070	.380	-.1833	.0366
		N1	C	.11000*	.03070	.050	.0001	.2199
			SC	.07000	.03070	.458	-.0399	.1799
			N2	.01667	.03070	1.000	-.0933	.1266
			N3	-.00333	.03070	1.000	-.1133	.1066
		N2	C	.09333	.03070	.124	-.0166	.2033
			SC	.05333	.03070	1.000	-.0566	.1633
			N1	-.01667	.03070	1.000	-.1266	.0933
			N3	-.02000	.03070	1.000	-.1299	.0899
		N3	C	.11333*	.03070	.042	.0034	.2233
			SC	.07333	.03070	.380	-.0366	.1833
			N1	.00333	.03070	1.000	-.1066	.1133
			N2	.02000	.03070	1.000	-.0899	.1299
	Dunnett t (2-sided)[b]	SC	C	.04000	.03070	.527	-.0487	.1287
		N1	C	.11000*	.03070	.016	.0213	.1987
		N2	C	.09333*	.03070	.039	.0046	.1821
		N3	C	.11333*	.03070	.014	.0246	.2021

*. The mean difference is significant at the 0.05 level.

b. Dunnett t-tests treat one group as a control, and compare all other groups against it.

Table S3. Post-hoc pairwise comparisons of proline and total phenols content across treatment groups using Bonferroni and Dunnett's tests.

Dependent Variable		(I) Variants	(J) Variants	Mean Difference (I-J)	Std. Error	Sig.	95% Confidence Interval	
							Lower Bound	Upper Bound
Proline	Bonferroni	C	SC	.23855	.22252	1.000	-.5584	1.0355
			N1	.38720	.22252	1.000	-.4097	1.1841
			N2	.12467	.22252	1.000	-.6723	.9216
			N3	-.14505	.22252	1.000	-.9420	.6519
		SC	C	-.23855	.22252	1.000	-1.0355	.5584
			N1	.14865	.22252	1.000	-.6483	.9456
			N2	-.11388	.22252	1.000	-.9108	.6830
			N3	-.38360	.22252	1.000	-1.1805	.4133
		N1	C	-.38720	.22252	1.000	-1.1841	.4097
			SC	-.14865	.22252	1.000	-.9456	.6483
			N2	-.26253	.22252	1.000	-1.0595	.5344
			N3	-.53225	.22252	.378	-1.3292	.2647
		N2	C	-.12467	.22252	1.000	-.9216	.6723
			SC	.11388	.22252	1.000	-.6830	.9108
			N1	.26253	.22252	1.000	-.5344	1.0595
			N3	-.26972	.22252	1.000	-1.0666	.5272
		N3	C	.14505	.22252	1.000	-.6519	.9420
			SC	.38360	.22252	1.000	-.4133	1.1805
			N1	.53225	.22252	.378	-.2647	1.3292
			N2	.26972	.22252	1.000	-.5272	1.0666
	Dunnett t (2-sided)[a]	SC	C	-.23855	.22252	.673	-.8817	.4046
		N1	C	-.38720	.22252	.300	-1.0304	.2560
		N2	C	-.12467	.22252	.944	-.7679	.5185
		N3	C	.14505	.22252	.910	-.4981	.7882
Phenols	Bonferroni	C	SC	-47.47475*	6.82099	.000	-71.9035	-23.0460
			N1	-10.77441	6.82099	1.000	-35.2031	13.6543
			N2	-4.71380	6.82099	1.000	-29.1425	19.7149
			N3	-8.75421	6.82099	1.000	-33.1829	15.6745
		SC	C	47.47475*	6.82099	.000	23.0460	71.9035
			N1	36.70034*	6.82099	.003	12.2716	61.1291
			N2	42.76094*	6.82099	.001	18.3322	67.1897
			N3	38.72054*	6.82099	.002	14.2918	63.1493
		N1	C	10.77441	6.82099	1.000	-13.6543	35.2031
			SC	-36.70034*	6.82099	.003	-61.1291	-12.2716
			N2	6.06061	6.82099	1.000	-18.3681	30.4893
			N3	2.02020	6.82099	1.000	-22.4085	26.4489
		N2	C	4.71380	6.82099	1.000	-19.7149	29.1425
			SC	-42.76094*	6.82099	.001	-67.1897	-18.3322
			N1	-6.06061	6.82099	1.000	-30.4893	18.3681
			N3	-4.04040	6.82099	1.000	-28.4691	20.3883
		N3	C	8.75421	6.82099	1.000	-15.6745	33.1829
			SC	-38.72054*	6.82099	.002	-63.1493	-14.2918
			N1	-2.02020	6.82099	1.000	-26.4489	22.4085
			N2	4.04040	6.82099	1.000	-20.3883	28.4691
	Dunnett t (2-sided)[a]	SC	C	47.47475*	6.82099	.000	27.7588	67.1907
		N1	C	10.77441	6.82099	.374	-8.9415	30.4903
		N2	C	4.71380	6.82099	.893	-15.0021	24.4297
		N3	C	8.75421	6.82099	.539	-10.9617	28.4701

*. The mean difference is significant at the 0.05 level.
a. Dunnett t-tests treat one group as a control, and compare all other groups against it.

Table S4. Post-hoc pairwise comparisons of assimilatory pigments content across treatment groups using Bonferroni and Dunnett's tests.

Dependent Variable		(I) Variants	(J) Variants	Mean Difference (I-J)	Std. Error	Sig.	95% Confidence Interval	
							Lower Bound	Upper Bound
Chlorophyll a	Bonferroni	C	SC	.41165	.29309	1.000	-.6380	1.4613
			N1	.01061	.29309	1.000	-1.0391	1.0603
			N2	.33365	.29309	1.000	-.7160	1.3833
			N3	-.24296	.29309	1.000	-1.2926	.8067
		SC	C	-.41165	.29309	1.000	-1.4613	.6380
			N1	-.40104	.29309	1.000	-1.4507	.6486
			N2	-.07799	.29309	1.000	-1.1277	.9717
			N3	-.65461	.29309	.496	-1.7043	.3951
		N1	C	-.01061	.29309	1.000	-1.0603	1.0391
			SC	.40104	.29309	1.000	-.6486	1.4507
			N2	.32304	.29309	1.000	-.7266	1.3727
			N3	-.25357	.29309	1.000	-1.3033	.7961
		N2	C	-.33365	.29309	1.000	-1.3833	.7160
			SC	.07799	.29309	1.000	-.9717	1.1277
			N1	-.32304	.29309	1.000	-1.3727	.7266
			N3	-.57661	.29309	.775	-1.6263	.4731
		N3	C	.24296	.29309	1.000	-.8067	1.2926
			SC	.65461	.29309	.496	-.3951	1.7043
			N1	.25357	.29309	1.000	-.7961	1.3033
			N2	.57661	.29309	.775	-.4731	1.6263
	Dunnett t (2-sided)[a]	SC	C	-.41165	.29309	.467	-1.2588	.4355
		N1	C	-.01061	.29309	1.000	-.8578	.8366
		N2	C	-.33365	.29309	.630	-1.1808	.5135
		N3	C	.24296	.29309	.822	-.6042	1.0901
Chlorophyll b	Bonferroni	C	SC	.21973	.24532	1.000	-.6589	1.0983
			N1	-.00190	.24532	1.000	-.8805	.8767
			N2	.16891	.24532	1.000	-.7097	1.0475
			N3	-.43387	.24532	1.000	-1.3125	.4447
		SC	C	-.21973	.24532	1.000	-1.0983	.6589
			N1	-.22163	.24532	1.000	-1.1002	.6570
			N2	-.05082	.24532	1.000	-.9294	.8278
			N3	-.65360	.24532	.237	-1.5322	.2250
		N1	C	.00190	.24532	1.000	-.8767	.8805
			SC	.22163	.24532	1.000	-.6570	1.1002
			N2	.17081	.24532	1.000	-.7078	1.0494
			N3	-.43197	.24532	1.000	-1.3106	.4466
		N2	C	-.16891	.24532	1.000	-1.0475	.7097
			SC	.05082	.24532	1.000	-.8278	.9294
			N1	-.17081	.24532	1.000	-1.0494	.7078
			N3	-.60278	.24532	.338	-1.4814	.2758
		N3	C	.43387	.24532	1.000	-.4447	1.3125
			SC	.65360	.24532	.237	-.2250	1.5322
			N1	.43197	.24532	1.000	-.4466	1.3106
			N2	.60278	.24532	.338	-.2758	1.4814
	Dunnett t (2-sided)[a]	SC	C	-.21973	.24532	.783	-.9288	.4894
		N1	C	.00190	.24532	1.000	-.7072	.7110

		N2	C	-.16891	.24532	.894	-.8780	.5402
		N3	C	.43387	.24532	.288	-.2752	1.1430
Carotenoids	Bonferroni	C	SC	.04013	.02293	1.000	-.0420	.1222
			N1	.03097	.02293	1.000	-.0511	.1131
			N2	.05107	.02293	.500	-.0310	.1332
			N3	.03673	.02293	1.000	-.0454	.1188
		SC	C	-.04013	.02293	1.000	-.1222	.0420
			N1	-.00915	.02293	1.000	-.0913	.0730
			N2	.01094	.02293	1.000	-.0712	.0931
			N3	-.00340	.02293	1.000	-.0855	.0787
		N1	C	-.03097	.02293	1.000	-.1131	.0511
			SC	.00915	.02293	1.000	-.0730	.0913
			N2	.02010	.02293	1.000	-.0620	.1022
			N3	.00576	.02293	1.000	-.0764	.0879
		N2	C	-.05107	.02293	.500	-.1332	.0310
			SC	-.01094	.02293	1.000	-.0931	.0712
			N1	-.02010	.02293	1.000	-.1022	.0620
			N3	-.01434	.02293	1.000	-.0965	.0678
		N3	C	-.03673	.02293	1.000	-.1188	.0454
			SC	.00340	.02293	1.000	-.0787	.0855
			N1	-.00576	.02293	1.000	-.0879	.0764
			N2	.01434	.02293	1.000	-.0678	.0965
	Dunnett t (2-sided)[a]	SC	C	-.04013	.02293	.296	-.1064	.0261
		N1	C	-.03097	.02293	.499	-.0972	.0353
		N2	C	-.05107	.02293	.145	-.1173	.0152
		N3	C	-.03673	.02293	.363	-.1030	.0295

a. Dunnett t-tests treat one group as a control, and compare all other groups against it.

Table S5. Post-hoc pairwise comparisons of cell viability across treatment groups using Bonferroni and Dunnett's tests.

	(I) Variants	(J) Variants	Mean Difference (I-J)	Std. Error	Sig.	95% Confidence Interval	
						Lower Bound	Upper Bound
Bonferroni	C	SC	-.01900	.00555	.065	-.0389	.0009
		N1	-.01000	.00555	1.000	-.0299	.0099
		N2	-.00100	.00555	1.000	-.0209	.0189
		N3	-.00067	.00555	1.000	-.0206	.0192
	SC	C	.01900	.00555	.065	-.0009	.0389
		N1	.00900	.00555	1.000	-.0109	.0289
		N2	.01800	.00555	.089	-.0019	.0379
		N3	.01833	.00555	.080	-.0016	.0382
	N1	C	.01000	.00555	1.000	-.0099	.0299
		SC	-.00900	.00555	1.000	-.0289	.0109
		N2	.00900	.00555	1.000	-.0109	.0289
		N3	.00933	.00555	1.000	-.0106	.0292
	N2	C	.00100	.00555	1.000	-.0189	.0209
		SC	-.01800	.00555	.089	-.0379	.0019
		N1	-.00900	.00555	1.000	-.0289	.0109
		N3	.00033	.00555	1.000	-.0196	.0202
	N3	C	.00067	.00555	1.000	-.0192	.0206
		SC	-.01833	.00555	.080	-.0382	.0016
		N1	-.00933	.00555	1.000	-.0292	.0106

		N2	-.00033	.00555	1.000	-.0202	.0196
Dunnett t (2-sided)[a]	SC	C	.01900*	.00555	.021	.0029	.0351
	N1	C	.01000	.00555	.275	-.0061	.0261
	N2	C	.00100	.00555	.999	-.0151	.0171
	N3	C	.00067	.00555	1.000	-.0154	.0167

*. The mean difference is significant at the 0.05 level.

a. Dunnett t-tests treat one group as a control, and compare all other groups against it.

Table S6. Post-hoc pairwise comparisons of mitotic index across treatment groups using Bonferroni and Dunnett's tests.

	(I) VAR00002	(J) VAR00002	Mean Difference (I-J)	Std. Error	Sig.	95% Confidence Interval	
						Lower Bound	Upper Bound
Bonferroni	DW24H	DW48H	1.48006	1.23763	1.000	-3.2922	6.2523
		SC24H	-1.79073	1.23763	1.000	-6.5630	2.9815
		SC48H	-.75115	1.23763	1.000	-5.5234	4.0211
		N1 24H	-1.03644	1.23763	1.000	-5.8087	3.7358
		N1 48H	-2.39454	1.23763	1.000	-7.1668	2.3777
		N2 24H	-2.27465	1.23763	1.000	-7.0469	2.4976
		N2 48H	-.90624	1.23763	1.000	-5.6785	3.8660
		N3 24H	-2.22528	1.23763	1.000	-6.9976	2.5470
		N3 48H	-3.26256	1.23763	.955	-8.0348	1.5097
		MMS 24H	3.24984	1.23763	.978	-1.5224	8.0221
		MMS 48H	3.31651	1.23763	.865	-1.4558	8.0888
	DW48H	DW24H	-1.48006	1.23763	1.000	-6.2523	3.2922
		SC24H	-3.27079	1.23763	.941	-8.0431	1.5015
		SC48H	-2.23121	1.23763	1.000	-7.0035	2.5411
		N1 24H	-2.51649	1.23763	1.000	-7.2888	2.2558
		N1 48H	-3.87459	1.23763	.300	-8.6469	.8977
		N2 24H	-3.75470	1.23763	.378	-8.5270	1.0176
		N2 48H	-2.38629	1.23763	1.000	-7.1586	2.3860
		N3 24H	-3.70533	1.23763	.416	-8.4776	1.0669
		N3 48H	-4.74261	1.23763	.053	-9.5149	.0297
		MMS 24H	1.76978	1.23763	1.000	-3.0025	6.5421
		MMS 48H	1.83645	1.23763	1.000	-2.9358	6.6087
	SC24H	DW24H	1.79073	1.23763	1.000	-2.9815	6.5630
		DW48H	3.27079	1.23763	.941	-1.5015	8.0431
		SC48H	1.03958	1.23763	1.000	-3.7327	5.8119
		N1 24H	.75430	1.23763	1.000	-4.0180	5.5266
		N1 48H	-.60380	1.23763	1.000	-5.3761	4.1685
		N2 24H	-.48391	1.23763	1.000	-5.2562	4.2884
		N2 48H	.88450	1.23763	1.000	-3.8878	5.6568
		N3 24H	-.43454	1.23763	1.000	-5.2068	4.3377
		N3 48H	-1.47182	1.23763	1.000	-6.2441	3.3005
		MMS 24H	5.04057*	1.23763	.029	.2683	9.8129
		MMS 48H	5.10724*	1.23763	.025	.3350	9.8795
	SC48H	DW24H	.75115	1.23763	1.000	-4.0211	5.5234
		DW48H	2.23121	1.23763	1.000	-2.5411	7.0035
		SC24H	-1.03958	1.23763	1.000	-5.8119	3.7327
		N1 24H	-.28528	1.23763	1.000	-5.0576	4.4870
		N1 48H	-1.64338	1.23763	1.000	-6.4157	3.1289
		N2 24H	-1.52349	1.23763	1.000	-6.2958	3.2488
		N2 48H	-.15509	1.23763	1.000	-4.9274	4.6172

	N3 24H	-1.47413	1.23763	1.000	-6.2464	3.2982
	N3 48H	-2.51141	1.23763	1.000	-7.2837	2.2609
	MMS 24H	4.00099	1.23763	.234	-.7713	8.7733
	MMS 48H	4.06766	1.23763	.205	-.7046	8.8399
N1 24H	DW24H	1.03644	1.23763	1.000	-3.7358	5.8087
	DW48H	2.51649	1.23763	1.000	-2.2558	7.2888
	SC24H	-.75430	1.23763	1.000	-5.5266	4.0180
	SC48H	.28528	1.23763	1.000	-4.4870	5.0576
	N1 48H	-1.35810	1.23763	1.000	-6.1304	3.4142
	N2 24H	-1.23821	1.23763	1.000	-6.0105	3.5341
	N2 48H	.13020	1.23763	1.000	-4.6421	4.9025
	N3 24H	-1.18884	1.23763	1.000	-5.9611	3.5834
	N3 48H	-2.22612	1.23763	1.000	-6.9984	2.5462
	MMS 24H	4.28628	1.23763	.133	-.4860	9.0586
	MMS 48H	4.35294	1.23763	.117	-.4193	9.1252
N1 48H	DW24H	2.39454	1.23763	1.000	-2.3777	7.1668
	DW48H	3.87459	1.23763	.300	-.8977	8.6469
	SC24H	.60380	1.23763	1.000	-4.1685	5.3761
	SC48H	1.64338	1.23763	1.000	-3.1289	6.4157
	N1 24H	1.35810	1.23763	1.000	-3.4142	6.1304
	N2 24H	.11989	1.23763	1.000	-4.6524	4.8922
	N2 48H	1.48830	1.23763	1.000	-3.2840	6.2606
	N3 24H	.16926	1.23763	1.000	-4.6030	4.9415
	N3 48H	-.86802	1.23763	1.000	-5.6403	3.9043
	MMS 24H	5.64438*	1.23763	.008	.8721	10.4167
	MMS 48H	5.71104*	1.23763	.007	.9388	10.4833
N2 24H	DW24H	2.27465	1.23763	1.000	-2.4976	7.0469
	DW48H	3.75470	1.23763	.378	-1.0176	8.5270
	SC24H	.48391	1.23763	1.000	-4.2884	5.2562
	SC48H	1.52349	1.23763	1.000	-3.2488	6.2958
	N1 24H	1.23821	1.23763	1.000	-3.5341	6.0105
	N1 48H	-.11989	1.23763	1.000	-4.8922	4.6524
	N2 48H	1.36841	1.23763	1.000	-3.4039	6.1407
	N3 24H	.04937	1.23763	1.000	-4.7229	4.8216
	N3 48H	-.98791	1.23763	1.000	-5.7602	3.7844
	MMS 24H	5.52448*	1.23763	.011	.7522	10.2968
	MMS 48H	5.59115*	1.23763	.009	.8189	10.3634
N2 48H	DW24H	.90624	1.23763	1.000	-3.8660	5.6785
	DW48H	2.38629	1.23763	1.000	-2.3860	7.1586
	SC24H	-.88450	1.23763	1.000	-5.6568	3.8878
	SC48H	.15509	1.23763	1.000	-4.6172	4.9274
	N1 24H	-.13020	1.23763	1.000	-4.9025	4.6421
	N1 48H	-1.48830	1.23763	1.000	-6.2606	3.2840
	N2 24H	-1.36841	1.23763	1.000	-6.1407	3.4039
	N3 24H	-1.31904	1.23763	1.000	-6.0913	3.4532
	N3 48H	-2.35632	1.23763	1.000	-7.1286	2.4160
	MMS 24H	4.15608	1.23763	.172	-.6162	8.9284
	MMS 48H	4.22274	1.23763	.151	-.5495	8.9950
N3 24H	DW24H	2.22528	1.23763	1.000	-2.5470	6.9976
	DW48H	3.70533	1.23763	.416	-1.0669	8.4776
	SC24H	.43454	1.23763	1.000	-4.3377	5.2068
	SC48H	1.47413	1.23763	1.000	-3.2982	6.2464
	N1 24H	1.18884	1.23763	1.000	-3.5834	5.9611
	N1 48H	-.16926	1.23763	1.000	-4.9415	4.6030
	N2 24H	-.04937	1.23763	1.000	-4.8216	4.7229

		N2 48H	1.31904	1.23763	1.000	-3.4532	6.0913
		N3 48H	-1.03728	1.23763	1.000	-5.8096	3.7350
		MMS 24H	5.47512*	1.23763	.012	.7028	10.2474
		MMS 48H	5.54178*	1.23763	.010	.7695	10.3141
	N3 48H	DW24H	3.26256	1.23763	.955	-1.5097	8.0348
		DW48H	4.74261	1.23763	.053	-.0297	9.5149
		SC24H	1.47182	1.23763	1.000	-3.3005	6.2441
		SC48H	2.51141	1.23763	1.000	-2.2609	7.2837
		N1 24H	2.22612	1.23763	1.000	-2.5462	6.9984
		N1 48H	.86802	1.23763	1.000	-3.9043	5.6403
		N2 24H	.98791	1.23763	1.000	-3.7844	5.7602
		N2 48H	2.35632	1.23763	1.000	-2.4160	7.1286
		N3 24H	1.03728	1.23763	1.000	-3.7350	5.8096
		MMS 24H	6.51240*	1.23763	.001	1.7401	11.2847
		MMS 48H	6.57906*	1.23763	.001	1.8068	11.3513
	MMS 24H	DW24H	-3.24984	1.23763	.978	-8.0221	1.5224
		DW48H	-1.76978	1.23763	1.000	-6.5421	3.0025
		SC24H	-5.04057*	1.23763	.029	-9.8129	-.2683
		SC48H	-4.00099	1.23763	.234	-8.7733	.7713
		N1 24H	-4.28628	1.23763	.133	-9.0586	.4860
		N1 48H	-5.64438*	1.23763	.008	-10.4167	-.8721
		N2 24H	-5.52448*	1.23763	.011	-10.2968	-.7522
		N2 48H	-4.15608	1.23763	.172	-8.9284	.6162
		N3 24H	-5.47512*	1.23763	.012	-10.2474	-.7028
		N3 48H	-6.51240*	1.23763	.001	-11.2847	-1.7401
		MMS 48H	.06667	1.23763	1.000	-4.7056	4.8389
	MMS 48H	DW24H	-3.31651	1.23763	.865	-8.0888	1.4558
		DW48H	-1.83645	1.23763	1.000	-6.6087	2.9358
		SC24H	-5.10724*	1.23763	.025	-9.8795	-.3350
		SC48H	-4.06766	1.23763	.205	-8.8399	.7046
		N1 24H	-4.35294	1.23763	.117	-9.1252	.4193
		N1 48H	-5.71104*	1.23763	.007	-10.4833	-.9388
		N2 24H	-5.59115*	1.23763	.009	-10.3634	-.8189
		N2 48H	-4.22274	1.23763	.151	-8.9950	.5495
		N3 24H	-5.54178*	1.23763	.010	-10.3141	-.7695
		N3 48H	-6.57906*	1.23763	.001	-11.3513	-1.8068
		MMS 24H	-.06667	1.23763	1.000	-4.8389	4.7056
Dunnett t (2-sided)[b]	DW24H	MMS 48H	3.31651	1.23763	.091	-.3564	6.9894
	DW48H	MMS 48H	1.83645	1.23763	.645	-1.8365	5.5094
	SC24H	MMS 48H	5.10724*	1.23763	.003	1.4343	8.7802
	SC48H	MMS 48H	4.06766*	1.23763	.025	.3947	7.7406
	N1 24H	MMS 48H	4.35294*	1.23763	.015	.6800	8.0259
	N1 48H	MMS 48H	5.71104*	1.23763	.001	2.0381	9.3840
	N2 24H	MMS 48H	5.59115*	1.23763	.001	1.9182	9.2641
	N2 48H	MMS 48H	4.22274*	1.23763	.019	.5498	7.8957
	N3 24H	MMS 48H	5.54178*	1.23763	.001	1.8689	9.2147
	N3 48H	MMS 48H	6.57906*	1.23763	.000	2.9062	10.2520
	MMS 24H	MMS 48H	.06667	1.23763	1.000	-3.6062	3.7396

*. The mean difference is significant at the 0.05 level.

b. Dunnett t-tests treat one group as a control, and compare all other groups against it.

Table S7. Post-hoc pairwise comparisons of prophase index across treatment groups using Bonferroni and Dunnett's tests.

	(I) VAR00004	(J) VAR00004		Std. Error	Sig.	95% Confidence Interval	

			Mean Difference (I-J)			Lower Bound	Upper Bound
Bonferroni	AD24H	AD48H	-29.48665	10.41661	.610	-69.6530	10.6797
		CS24H	-4.41685	10.41661	1.000	-44.5832	35.7495
		CS48H	-1.96241	10.41661	1.000	-42.1288	38.2040
		N1 24H	4.87861	10.41661	1.000	-35.2878	45.0450
		N1 48H	-3.81850	10.41661	1.000	-43.9849	36.3479
		N2 24H	14.03709	10.41661	1.000	-26.1293	54.2035
		N2 48H	11.06098	10.41661	1.000	-29.1054	51.2274
		N3 24H	2.57214	10.41661	1.000	-37.5942	42.7385
		N3 48H	12.85449	10.41661	1.000	-27.3119	53.0209
		MMS 24H	26.19192	10.41661	1.000	-13.9745	66.3583
		MMS 48H	42.85859*	10.41661	.026	2.6922	83.0250
	AD48H	AD24H	29.48665	10.41661	.610	-10.6797	69.6530
		CS24H	25.06980	10.41661	1.000	-15.0966	65.2362
		CS48H	27.52423	10.41661	.942	-12.6422	67.6906
		N1 24H	34.36525	10.41661	.199	-5.8011	74.5316
		N1 48H	25.66815	10.41661	1.000	-14.4982	65.8345
		N2 24H	43.52373*	10.41661	.022	3.3573	83.6901
		N2 48H	40.54763*	10.41661	.046	.3812	80.7140
		N3 24H	32.05879	10.41661	.340	-8.1076	72.2252
		N3 48H	42.34114*	10.41661	.030	2.1747	82.5075
		MMS 24H	55.67857*	10.41661	.001	15.5122	95.8450
		MMS 48H	72.34524*	10.41661	.000	32.1788	112.5116
	CS24H	AD24H	4.41685	10.41661	1.000	-35.7495	44.5832
		AD48H	-25.06980	10.41661	1.000	-65.2362	15.0966
		CS48H	2.45444	10.41661	1.000	-37.7120	42.6208
		N1 24H	9.29546	10.41661	1.000	-30.8709	49.4618
		N1 48H	.59835	10.41661	1.000	-39.5680	40.7647
		N2 24H	18.45394	10.41661	1.000	-21.7125	58.6203
		N2 48H	15.47783	10.41661	1.000	-24.6886	55.6442
		N3 24H	6.98899	10.41661	1.000	-33.1774	47.1554
		N3 48H	17.27134	10.41661	1.000	-22.8951	57.4377
		MMS 24H	30.60877	10.41661	.474	-9.5576	70.7752
		MMS 48H	47.27544*	10.41661	.009	7.1090	87.4418
	CS48H	AD24H	1.96241	10.41661	1.000	-38.2040	42.1288
		AD48H	-27.52423	10.41661	.942	-67.6906	12.6422
		CS24H	-2.45444	10.41661	1.000	-42.6208	37.7120
		N1 24H	6.84102	10.41661	1.000	-33.3254	47.0074
		N1 48H	-1.85608	10.41661	1.000	-42.0225	38.3103
		N2 24H	15.99950	10.41661	1.000	-24.1669	56.1659
		N2 48H	13.02339	10.41661	1.000	-27.1430	53.1898
		N3 24H	4.53456	10.41661	1.000	-35.6318	44.7009
		N3 48H	14.81690	10.41661	1.000	-25.3495	54.9833
		MMS 24H	28.15434	10.41661	.820	-12.0121	68.3207
		MMS 48H	44.82100*	10.41661	.016	4.6546	84.9874
	N1 24H	AD24H	-4.87861	10.41661	1.000	-45.0450	35.2878
		AD48H	-34.36525	10.41661	.199	-74.5316	5.8011
		CS24H	-9.29546	10.41661	1.000	-49.4618	30.8709
		CS48H	-6.84102	10.41661	1.000	-47.0074	33.3254
		N1 48H	-8.69710	10.41661	1.000	-48.8635	31.4693
		N2 24H	9.15848	10.41661	1.000	-31.0079	49.3249
		N2 48H	6.18237	10.41661	1.000	-33.9840	46.3488
		N3 24H	-2.30646	10.41661	1.000	-42.4729	37.8599
		N3 48H	7.97588	10.41661	1.000	-32.1905	48.1423

		MMS 24H	21.31332	10.41661	1.000	-18.8531	61.4797
		MMS 48H	37.97998	10.41661	.085	-2.1864	78.1464
	N1 48H	AD24H	3.81850	10.41661	1.000	-36.3479	43.9849
		AD48H	-25.66815	10.41661	1.000	-65.8345	14.4982
		CS24H	-.59835	10.41661	1.000	-40.7647	39.5680
		CS48H	1.85608	10.41661	1.000	-38.3103	42.0225
		N1 24H	8.69710	10.41661	1.000	-31.4693	48.8635
		N2 24H	17.85558	10.41661	1.000	-22.3108	58.0220
		N2 48H	14.87948	10.41661	1.000	-25.2869	55.0459
		N3 24H	6.39064	10.41661	1.000	-33.7758	46.5570
		N3 48H	16.67299	10.41661	1.000	-23.4934	56.8394
		MMS 24H	30.01042	10.41661	.542	-10.1560	70.1768
		MMS 48H	46.67709*	10.41661	.010	6.5107	86.8435
	N2 24H	AD24H	-14.03709	10.41661	1.000	-54.2035	26.1293
		AD48H	-43.52373*	10.41661	.022	-83.6901	-3.3573
		CS24H	-18.45394	10.41661	1.000	-58.6203	21.7125
		CS48H	-15.99950	10.41661	1.000	-56.1659	24.1669
		N1 24H	-9.15848	10.41661	1.000	-49.3249	31.0079
		N1 48H	-17.85558	10.41661	1.000	-58.0220	22.3108
		N2 48H	-2.97611	10.41661	1.000	-43.1425	37.1903
		N3 24H	-11.46494	10.41661	1.000	-51.6313	28.7014
		N3 48H	-1.18260	10.41661	1.000	-41.3490	38.9838
		MMS 24H	12.15484	10.41661	1.000	-28.0116	52.3212
		MMS 48H	28.82150	10.41661	.708	-11.3449	68.9879
	N2 48H	AD24H	-11.06098	10.41661	1.000	-51.2274	29.1054
		AD48H	-40.54763*	10.41661	.046	-80.7140	-.3812
		CS24H	-15.47783	10.41661	1.000	-55.6442	24.6886
		CS48H	-13.02339	10.41661	1.000	-53.1898	27.1430
		N1 24H	-6.18237	10.41661	1.000	-46.3488	33.9840
		N1 48H	-14.87948	10.41661	1.000	-55.0459	25.2869
		N2 24H	2.97611	10.41661	1.000	-37.1903	43.1425
		N3 24H	-8.48884	10.41661	1.000	-48.6552	31.6776
		N3 48H	1.79351	10.41661	1.000	-38.3729	41.9599
		MMS 24H	15.13094	10.41661	1.000	-25.0354	55.2973
		MMS 48H	31.79761	10.41661	.361	-8.3688	71.9640
	N3 24H	AD24H	-2.57214	10.41661	1.000	-42.7385	37.5942
		AD48H	-32.05879	10.41661	.340	-72.2252	8.1076
		CS24H	-6.98899	10.41661	1.000	-47.1554	33.1774
		CS48H	-4.53456	10.41661	1.000	-44.7009	35.6318
		N1 24H	2.30646	10.41661	1.000	-37.8599	42.4729
		N1 48H	-6.39064	10.41661	1.000	-46.5570	33.7758
		N2 24H	11.46494	10.41661	1.000	-28.7014	51.6313
		N2 48H	8.48884	10.41661	1.000	-31.6776	48.6552
		N3 48H	10.28235	10.41661	1.000	-29.8840	50.4487
		MMS 24H	23.61978	10.41661	1.000	-16.5466	63.7862
		MMS 48H	40.28645*	10.41661	.049	.1201	80.4528
	N3 48H	AD24H	-12.85449	10.41661	1.000	-53.0209	27.3119
		AD48H	-42.34114*	10.41661	.030	-82.5075	-2.1747
		CS24H	-17.27134	10.41661	1.000	-57.4377	22.8951
		CS48H	-14.81690	10.41661	1.000	-54.9833	25.3495
		N1 24H	-7.97588	10.41661	1.000	-48.1423	32.1905
		N1 48H	-16.67299	10.41661	1.000	-56.8394	23.4934
		N2 24H	1.18260	10.41661	1.000	-38.9838	41.3490
		N2 48H	-1.79351	10.41661	1.000	-41.9599	38.3729
		N3 24H	-10.28235	10.41661	1.000	-50.4487	29.8840

	(I)	(J)	Mean Difference (I-J)	Std. Error	Sig.	Lower Bound	Upper Bound
		MMS 24H	13.33743	10.41661	1.000	-26.8290	53.5038
		MMS 48H	30.00410	10.41661	.543	-10.1623	70.1705
	MMS 24H	AD24H	-26.19192	10.41661	1.000	-66.3583	13.9745
		AD48H	-55.67857*	10.41661	.001	-95.8450	-15.5122
		CS24H	-30.60877	10.41661	.474	-70.7752	9.5576
		CS48H	-28.15434	10.41661	.820	-68.3207	12.0121
		N1 24H	-21.31332	10.41661	1.000	-61.4797	18.8531
		N1 48H	-30.01042	10.41661	.542	-70.1768	10.1560
		N2 24H	-12.15484	10.41661	1.000	-52.3212	28.0116
		N2 48H	-15.13094	10.41661	1.000	-55.2973	25.0354
		N3 24H	-23.61978	10.41661	1.000	-63.7862	16.5466
		N3 48H	-13.33743	10.41661	1.000	-53.5038	26.8290
		MMS 48H	16.66667	10.41661	1.000	-23.4997	56.8331
	MMS 48H	AD24H	-42.85859*	10.41661	.026	-83.0250	-2.6922
		AD48H	-72.34524*	10.41661	.000	-112.5116	-32.1788
		CS24H	-47.27544*	10.41661	.009	-87.4418	-7.1090
		CS48H	-44.82100*	10.41661	.016	-84.9874	-4.6546
		N1 24H	-37.97998	10.41661	.085	-78.1464	2.1864
		N1 48H	-46.67709*	10.41661	.010	-86.8435	-6.5107
		N2 24H	-28.82150	10.41661	.708	-68.9879	11.3449
		N2 48H	-31.79761	10.41661	.361	-71.9640	8.3688
		N3 24H	-40.28645*	10.41661	.049	-80.4528	-.1201
		N3 48H	-30.00410	10.41661	.543	-70.1705	10.1623
		MMS 24H	-16.66667	10.41661	1.000	-56.8331	23.4997
Dunnett t (2-sided)[b]	AD24H	MMS 48H	42.85859*	10.41661	.003	11.9452	73.7720
	AD48H	MMS 48H	72.34524*	10.41661	.000	41.4318	103.2587
	CS24H	MMS 48H	47.27544*	10.41661	.001	16.3620	78.1889
	CS48H	MMS 48H	44.82100*	10.41661	.002	13.9076	75.7344
	N1 24H	MMS 48H	37.97998*	10.41661	.011	7.0666	68.8934
	N1 48H	MMS 48H	46.67709*	10.41661	.001	15.7637	77.5905
	N2 24H	MMS 48H	28.82150	10.41661	.076	-2.0919	59.7349
	N2 48H	MMS 48H	31.79761*	10.41661	.042	.8842	62.7110
	N3 24H	MMS 48H	40.28645*	10.41661	.006	9.3730	71.1999
	N3 48H	MMS 48H	30.00410	10.41661	.060	-.9093	60.9175
	MMS 24H	MMS 48H	16.66667	10.41661	.564	-14.2468	47.5801

*. The mean difference is significant at the 0.05 level.

b. Dunnett t-tests treat one group as a control, and compare all other groups against it.

Table S8. Post-hoc pairwise comparisons of metaphase index across treatment groups using Bonferroni and Dunnett's tests.

	(I) VAR00006	(J) VAR00006	Mean Difference (I-J)	Std. Error	Sig.	95% Confidence Interval	
						Lower Bound	Upper Bound
Bonferroni	AD24H	AD48H	7.23081	5.78326	1.000	-15.0694	29.5310
		CS24H	8.95680	5.78326	1.000	-13.3434	31.2570
		CS48H	-8.90369	5.78326	1.000	-31.2039	13.3965
		N1 24H	-6.10101	5.78326	1.000	-28.4012	16.1992
		N1 48H	-1.11587	5.78326	1.000	-23.4161	21.1843
		N2 24H	-2.10152	5.78326	1.000	-24.4017	20.1987
		N2 48H	-15.20568	5.78326	.970	-37.5059	7.0945
		N3 24H	.60472	5.78326	1.000	-21.6955	22.9049
		N3 48H	-9.80314	5.78326	1.000	-32.1034	12.4971
		MMS 24H	25.37366*	5.78326	.013	3.0734	47.6739
		MMS 48H	25.37366*	5.78326	.013	3.0734	47.6739

AD48H	AD24H	-7.23081	5.78326	1.000	-29.5310	15.0694
	CS24H	1.72599	5.78326	1.000	-20.5742	24.0262
	CS48H	-16.13450	5.78326	.671	-38.4347	6.1657
	N1 24H	-13.33182	5.78326	1.000	-35.6320	8.9684
	N1 48H	-8.34668	5.78326	1.000	-30.6469	13.9535
	N2 24H	-9.33233	5.78326	1.000	-31.6325	12.9679
	N2 48H	-22.43649*	5.78326	.047	-44.7367	-.1363
	N3 24H	-6.62609	5.78326	1.000	-28.9263	15.6741
	N3 48H	-17.03394	5.78326	.466	-39.3342	5.2663
	MMS 24H	18.14286	5.78326	.295	-4.1574	40.4431
	MMS 48H	18.14286	5.78326	.295	-4.1574	40.4431
CS24H	AD24H	-8.95680	5.78326	1.000	-31.2570	13.3434
	AD48H	-1.72599	5.78326	1.000	-24.0262	20.5742
	CS48H	-17.86049	5.78326	.332	-40.1607	4.4397
	N1 24H	-15.05781	5.78326	1.000	-37.3580	7.2424
	N1 48H	-10.07267	5.78326	1.000	-32.3729	12.2275
	N2 24H	-11.05832	5.78326	1.000	-33.3585	11.2419
	N2 48H	-24.16249*	5.78326	.022	-46.4627	-1.8623
	N3 24H	-8.35209	5.78326	1.000	-30.6523	13.9481
	N3 48H	-18.75994	5.78326	.228	-41.0602	3.5403
	MMS 24H	16.41686	5.78326	.599	-5.8834	38.7171
	MMS 48H	16.41686	5.78326	.599	-5.8834	38.7171
CS48H	AD24H	8.90369	5.78326	1.000	-13.3965	31.2039
	AD48H	16.13450	5.78326	.671	-6.1657	38.4347
	CS24H	17.86049	5.78326	.332	-4.4397	40.1607
	N1 24H	2.80268	5.78326	1.000	-19.4975	25.1029
	N1 48H	7.78782	5.78326	1.000	-14.5124	30.0880
	N2 24H	6.80217	5.78326	1.000	-15.4980	29.1024
	N2 48H	-6.30199	5.78326	1.000	-28.6022	15.9982
	N3 24H	9.50841	5.78326	1.000	-12.7918	31.8086
	N3 48H	-.89944	5.78326	1.000	-23.1997	21.4008
	MMS 24H	34.27736*	5.78326	.000	11.9771	56.5776
	MMS 48H	34.27736*	5.78326	.000	11.9771	56.5776
N1 24H	AD24H	6.10101	5.78326	1.000	-16.1992	28.4012
	AD48H	13.33182	5.78326	1.000	-8.9684	35.6320
	CS24H	15.05781	5.78326	1.000	-7.2424	37.3580
	CS48H	-2.80268	5.78326	1.000	-25.1029	19.4975
	N1 48H	4.98514	5.78326	1.000	-17.3151	27.2854
	N2 24H	3.99949	5.78326	1.000	-18.3007	26.2997
	N2 48H	-9.10468	5.78326	1.000	-31.4049	13.1955
	N3 24H	6.70572	5.78326	1.000	-15.5945	29.0059
	N3 48H	-3.70213	5.78326	1.000	-26.0023	18.5981
	MMS 24H	31.47467*	5.78326	.001	9.1745	53.7749
	MMS 48H	31.47467*	5.78326	.001	9.1745	53.7749
N1 48H	AD24H	1.11587	5.78326	1.000	-21.1843	23.4161
	AD48H	8.34668	5.78326	1.000	-13.9535	30.6469
	CS24H	10.07267	5.78326	1.000	-12.2275	32.3729
	CS48H	-7.78782	5.78326	1.000	-30.0880	14.5124
	N1 24H	-4.98514	5.78326	1.000	-27.2854	17.3151
	N2 24H	-.98565	5.78326	1.000	-23.2859	21.3146
	N2 48H	-14.08982	5.78326	1.000	-36.3900	8.2104
	N3 24H	1.72058	5.78326	1.000	-20.5796	24.0208
	N3 48H	-8.68727	5.78326	1.000	-30.9875	13.6129
	MMS 24H	26.48953*	5.78326	.008	4.1893	48.7897
	MMS 48H	26.48953*	5.78326	.008	4.1893	48.7897

N2 24H	AD24H	2.10152	5.78326	1.000	-20.1987	24.4017
	AD48H	9.33233	5.78326	1.000	-12.9679	31.6325
	CS24H	11.05832	5.78326	1.000	-11.2419	33.3585
	CS48H	-6.80217	5.78326	1.000	-29.1024	15.4980
	N1 24H	-3.99949	5.78326	1.000	-26.2997	18.3007
	N1 48H	.98565	5.78326	1.000	-21.3146	23.2859
	N2 48H	-13.10416	5.78326	1.000	-35.4044	9.1961
	N3 24H	2.70624	5.78326	1.000	-19.5940	25.0065
	N3 48H	-7.70162	5.78326	1.000	-30.0018	14.5986
	MMS 24H	27.47518*	5.78326	.005	5.1750	49.7754
	MMS 48H	27.47518*	5.78326	.005	5.1750	49.7754
N2 48H	AD24H	15.20568	5.78326	.970	-7.0945	37.5059
	AD48H	22.43649*	5.78326	.047	.1363	44.7367
	CS24H	24.16249*	5.78326	.022	1.8623	46.4627
	CS48H	6.30199	5.78326	1.000	-15.9982	28.6022
	N1 24H	9.10468	5.78326	1.000	-13.1955	31.4049
	N1 48H	14.08982	5.78326	1.000	-8.2104	36.3900
	N2 24H	13.10416	5.78326	1.000	-9.1961	35.4044
	N3 24H	15.81040	5.78326	.764	-6.4898	38.1106
	N3 48H	5.40255	5.78326	1.000	-16.8977	27.7028
	MMS 24H	40.57935*	5.78326	.000	18.2791	62.8796
	MMS 48H	40.57935*	5.78326	.000	18.2791	62.8796
N3 24H	AD24H	-.60472	5.78326	1.000	-22.9049	21.6955
	AD48H	6.62609	5.78326	1.000	-15.6741	28.9263
	CS24H	8.35209	5.78326	1.000	-13.9481	30.6523
	CS48H	-9.50841	5.78326	1.000	-31.8086	12.7918
	N1 24H	-6.70572	5.78326	1.000	-29.0059	15.5945
	N1 48H	-1.72058	5.78326	1.000	-24.0208	20.5796
	N2 24H	-2.70624	5.78326	1.000	-25.0065	19.5940
	N2 48H	-15.81040	5.78326	.764	-38.1106	6.4898
	N3 48H	-10.40785	5.78326	1.000	-32.7081	11.8924
	MMS 24H	24.76895*	5.78326	.017	2.4687	47.0692
	MMS 48H	24.76895*	5.78326	.017	2.4687	47.0692
N3 48H	AD24H	9.80314	5.78326	1.000	-12.4971	32.1034
	AD48H	17.03394	5.78326	.466	-5.2663	39.3342
	CS24H	18.75994	5.78326	.228	-3.5403	41.0602
	CS48H	.89944	5.78326	1.000	-21.4008	23.1997
	N1 24H	3.70213	5.78326	1.000	-18.5981	26.0023
	N1 48H	8.68727	5.78326	1.000	-13.6129	30.9875
	N2 24H	7.70162	5.78326	1.000	-14.5986	30.0018
	N2 48H	-5.40255	5.78326	1.000	-27.7028	16.8977
	N3 24H	10.40785	5.78326	1.000	-11.8924	32.7081
	MMS 24H	35.17680*	5.78326	.000	12.8766	57.4770
	MMS 48H	35.17680*	5.78326	.000	12.8766	57.4770
MMS 24H	AD24H	-25.37366*	5.78326	.013	-47.6739	-3.0734
	AD48H	-18.14286	5.78326	.295	-40.4431	4.1574
	CS24H	-16.41686	5.78326	.599	-38.7171	5.8834
	CS48H	-34.27736*	5.78326	.000	-56.5776	-11.9771
	N1 24H	-31.47467*	5.78326	.001	-53.7749	-9.1745
	N1 48H	-26.48953*	5.78326	.008	-48.7897	-4.1893
	N2 24H	-27.47518*	5.78326	.005	-49.7754	-5.1750
	N2 48H	-40.57935*	5.78326	.000	-62.8796	-18.2791
	N3 24H	-24.76895*	5.78326	.017	-47.0692	-2.4687
	N3 48H	-35.17680*	5.78326	.000	-57.4770	-12.8766
	MMS 48H	.00000	5.78326	1.000	-22.3002	22.3002

	MMS 48H	AD24H	-25.37366*	5.78326	.013	-47.6739	-3.0734
		AD48H	-18.14286	5.78326	.295	-40.4431	4.1574
		CS24H	-16.41686	5.78326	.599	-38.7171	5.8834
		CS48H	-34.27736*	5.78326	.000	-56.5776	-11.9771
		N1 24H	-31.47467*	5.78326	.001	-53.7749	-9.1745
		N1 48H	-26.48953*	5.78326	.008	-48.7897	-4.1893
		N2 24H	-27.47518*	5.78326	.005	-49.7754	-5.1750
		N2 48H	-40.57935*	5.78326	.000	-62.8796	-18.2791
		N3 24H	-24.76895*	5.78326	.017	-47.0692	-2.4687
		N3 48H	-35.17680*	5.78326	.000	-57.4770	-12.8766
		MMS 24H	.00000	5.78326	1.000	-22.3002	22.3002
Dunnett t (2-sided)[b]	AD24H	MMS 48H	25.37366*	5.78326	.002	8.2107	42.5367
	AD48H	MMS 48H	18.14286*	5.78326	.034	.9798	35.3059
	CS24H	MMS 48H	16.41686	5.78326	.066	-.7461	33.5799
	CS48H	MMS 48H	34.27736*	5.78326	.000	17.1143	51.4404
	N1 24H	MMS 48H	31.47467*	5.78326	.000	14.3117	48.6377
	N1 48H	MMS 48H	26.48953*	5.78326	.001	9.3265	43.6525
	N2 24H	MMS 48H	27.47518*	5.78326	.001	10.3122	44.6382
	N2 48H	MMS 48H	40.57935*	5.78326	.000	23.4163	57.7424
	N3 24H	MMS 48H	24.76895*	5.78326	.002	7.6059	41.9320
	N3 48H	MMS 48H	35.17680*	5.78326	.000	18.0138	52.3398
	MMS 24H	MMS 48H	.00000	5.78326	1.000	-17.1630	17.1630

*. The mean difference is significant at the 0.05 level.

b. Dunnett t-tests treat one group as a control, and compare all other groups against it.

Table S9. Post-hoc pairwise comparisons of anaphase index across treatment groups using Bonferroni and Dunnett's tests.

	(I) VAR00008	(J) VAR00008	Mean Difference (I-J)	Std. Error	Sig.	95% Confidence Interval	
						Lower Bound	Upper Bound
Bonferroni	AD24H	AD48H	11.42767	4.72772	1.000	-6.8024	29.6577
		CS24H	-7.29447	4.72772	1.000	-25.5245	10.9356
		CS48H	1.94452	4.72772	1.000	-16.2856	20.1746
		N1 24H	-.45959	4.72772	1.000	-18.6897	17.7705
		N1 48H	-.66821	4.72772	1.000	-18.8983	17.5619
		N2 24H	-7.83263	4.72772	1.000	-26.0627	10.3974
		N2 48H	.53140	4.72772	1.000	-17.6987	18.7615
		N3 24H	-.79467	4.72772	1.000	-19.0247	17.4354
		N3 48H	-3.70992	4.72772	1.000	-21.9400	14.5202
		MMS 24H	13.51100	4.72772	.573	-4.7191	31.7411
		MMS 48H	13.51100	4.72772	.573	-4.7191	31.7411
	AD48H	AD24H	-11.42767	4.72772	1.000	-29.6577	6.8024
		CS24H	-18.72213*	4.72772	.038	-36.9522	-.4921
		CS48H	-9.48315	4.72772	1.000	-27.7132	8.7469
		N1 24H	-11.88725	4.72772	1.000	-30.1173	6.3428
		N1 48H	-12.09588	4.72772	1.000	-30.3260	6.1342
		N2 24H	-19.26030*	4.72772	.029	-37.4904	-1.0302
		N2 48H	-10.89627	4.72772	1.000	-29.1263	7.3338
		N3 24H	-12.22234	4.72772	1.000	-30.4524	6.0077
		N3 48H	-15.13758	4.72772	.252	-33.3677	3.0925
		MMS 24H	2.08333	4.72772	1.000	-16.1467	20.3134
		MMS 48H	2.08333	4.72772	1.000	-16.1467	20.3134

	CS24H	AD24H	7.29447	4.72772	1.000	-10.9356	25.5245
		AD48H	18.72213*	4.72772	.038	.4921	36.9522
		CS48H	9.23898	4.72772	1.000	-8.9911	27.4691
		N1 24H	6.83488	4.72772	1.000	-11.3952	25.0650
		N1 48H	6.62625	4.72772	1.000	-11.6038	24.8563
		N2 24H	-.53816	4.72772	1.000	-18.7682	17.6919
		N2 48H	7.82587	4.72772	1.000	-10.4042	26.0559
		N3 24H	6.49980	4.72772	1.000	-11.7303	24.7299
		N3 48H	3.58455	4.72772	1.000	-14.6455	21.8146
		MMS 24H	20.80547*	4.72772	.013	2.5754	39.0355
		MMS 48H	20.80547*	4.72772	.013	2.5754	39.0355
	CS48H	AD24H	-1.94452	4.72772	1.000	-20.1746	16.2856
		AD48H	9.48315	4.72772	1.000	-8.7469	27.7132
		CS24H	-9.23898	4.72772	1.000	-27.4691	8.9911
		N1 24H	-2.40410	4.72772	1.000	-20.6342	15.8260
		N1 48H	-2.61273	4.72772	1.000	-20.8428	15.6173
		N2 24H	-9.77715	4.72772	1.000	-28.0072	8.4529
		N2 48H	-1.41312	4.72772	1.000	-19.6432	16.8170
		N3 24H	-2.73919	4.72772	1.000	-20.9693	15.4909
		N3 48H	-5.65443	4.72772	1.000	-23.8845	12.5756
		MMS 24H	11.56648	4.72772	1.000	-6.6636	29.7966
		MMS 48H	11.56648	4.72772	1.000	-6.6636	29.7966
	N1 24H	AD24H	.45959	4.72772	1.000	-17.7705	18.6897
		AD48H	11.88725	4.72772	1.000	-6.3428	30.1173
		CS24H	-6.83488	4.72772	1.000	-25.0650	11.3952
		CS48H	2.40410	4.72772	1.000	-15.8260	20.6342
		N1 48H	-.20863	4.72772	1.000	-18.4387	18.0214
		N2 24H	-7.37304	4.72772	1.000	-25.6031	10.8570
		N2 48H	.99099	4.72772	1.000	-17.2391	19.2211
		N3 24H	-.33508	4.72772	1.000	-18.5652	17.8950
		N3 48H	-3.25033	4.72772	1.000	-21.4804	14.9797
		MMS 24H	13.97059	4.72772	.456	-4.2595	32.2007
		MMS 48H	13.97059	4.72772	.456	-4.2595	32.2007
	N1 48H	AD24H	.66821	4.72772	1.000	-17.5619	18.8983
		AD48H	12.09588	4.72772	1.000	-6.1342	30.3260
		CS24H	-6.62625	4.72772	1.000	-24.8563	11.6038
		CS48H	2.61273	4.72772	1.000	-15.6173	20.8428
		N1 24H	.20863	4.72772	1.000	-18.0214	18.4387
		N2 24H	-7.16441	4.72772	1.000	-25.3945	11.0657
		N2 48H	1.19962	4.72772	1.000	-17.0305	19.4297
		N3 24H	-.12645	4.72772	1.000	-18.3565	18.1036
		N3 48H	-3.04170	4.72772	1.000	-21.2718	15.1884
		MMS 24H	14.17922	4.72772	.410	-4.0509	32.4093
		MMS 48H	14.17922	4.72772	.410	-4.0509	32.4093
	N2 24H	AD24H	7.83263	4.72772	1.000	-10.3974	26.0627
		AD48H	19.26030*	4.72772	.029	1.0302	37.4904
		CS24H	.53816	4.72772	1.000	-17.6919	18.7682
		CS48H	9.77715	4.72772	1.000	-8.4529	28.0072
		N1 24H	7.37304	4.72772	1.000	-10.8570	25.6031
		N1 48H	7.16441	4.72772	1.000	-11.0657	25.3945
		N2 48H	8.36403	4.72772	1.000	-9.8660	26.5941
		N3 24H	7.03796	4.72772	1.000	-11.1921	25.2680
		N3 48H	4.12271	4.72772	1.000	-14.1074	22.3528
		MMS 24H	21.34363*	4.72772	.009	3.1136	39.5737
		MMS 48H	21.34363*	4.72772	.009	3.1136	39.5737

N2 48H	AD24H	-.53140	4.72772	1.000	-18.7615	17.6987
	AD48H	10.89627	4.72772	1.000	-7.3338	29.1263
	CS24H	-7.82587	4.72772	1.000	-26.0559	10.4042
	CS48H	1.41312	4.72772	1.000	-16.8170	19.6432
	N1 24H	-.99099	4.72772	1.000	-19.2211	17.2391
	N1 48H	-1.19962	4.72772	1.000	-19.4297	17.0305
	N2 24H	-8.36403	4.72772	1.000	-26.5941	9.8660
	N3 24H	-1.32607	4.72772	1.000	-19.5561	16.9040
	N3 48H	-4.24132	4.72772	1.000	-22.4714	13.9888
	MMS 24H	12.97960	4.72772	.744	-5.2505	31.2097
	MMS 48H	12.97960	4.72772	.744	-5.2505	31.2097
N3 24H	AD24H	.79467	4.72772	1.000	-17.4354	19.0247
	AD48H	12.22234	4.72772	1.000	-6.0077	30.4524
	CS24H	-6.49980	4.72772	1.000	-24.7299	11.7303
	CS48H	2.73919	4.72772	1.000	-15.4909	20.9693
	N1 24H	.33508	4.72772	1.000	-17.8950	18.5652
	N1 48H	.12645	4.72772	1.000	-18.1036	18.3565
	N2 24H	-7.03796	4.72772	1.000	-25.2680	11.1921
	N2 48H	1.32607	4.72772	1.000	-16.9040	19.5561
	N3 48H	-2.91525	4.72772	1.000	-21.1453	15.3148
	MMS 24H	14.30567	4.72772	.385	-3.9244	32.5357
	MMS 48H	14.30567	4.72772	.385	-3.9244	32.5357
N3 48H	AD24H	3.70992	4.72772	1.000	-14.5202	21.9400
	AD48H	15.13758	4.72772	.252	-3.0925	33.3677
	CS24H	-3.58455	4.72772	1.000	-21.8146	14.6455
	CS48H	5.65443	4.72772	1.000	-12.5756	23.8845
	N1 24H	3.25033	4.72772	1.000	-14.9797	21.4804
	N1 48H	3.04170	4.72772	1.000	-15.1884	21.2718
	N2 24H	-4.12271	4.72772	1.000	-22.3528	14.1074
	N2 48H	4.24132	4.72772	1.000	-13.9888	22.4714
	N3 24H	2.91525	4.72772	1.000	-15.3148	21.1453
	MMS 24H	17.22092	4.72772	.085	-1.0092	35.4510
	MMS 48H	17.22092	4.72772	.085	-1.0092	35.4510
MMS 24H	AD24H	-13.51100	4.72772	.573	-31.7411	4.7191
	AD48H	-2.08333	4.72772	1.000	-20.3134	16.1467
	CS24H	-20.80547*	4.72772	.013	-39.0355	-2.5754
	CS48H	-11.56648	4.72772	1.000	-29.7966	6.6636
	N1 24H	-13.97059	4.72772	.456	-32.2007	4.2595
	N1 48H	-14.17922	4.72772	.410	-32.4093	4.0509
	N2 24H	-21.34363*	4.72772	.009	-39.5737	-3.1136
	N2 48H	-12.97960	4.72772	.744	-31.2097	5.2505
	N3 24H	-14.30567	4.72772	.385	-32.5357	3.9244
	N3 48H	-17.22092	4.72772	.085	-35.4510	1.0092
	MMS 48H	.00000	4.72772	1.000	-18.2301	18.2301
MMS 48H	AD24H	-13.51100	4.72772	.573	-31.7411	4.7191
	AD48H	-2.08333	4.72772	1.000	-20.3134	16.1467
	CS24H	-20.80547*	4.72772	.013	-39.0355	-2.5754
	CS48H	-11.56648	4.72772	1.000	-29.7966	6.6636
	N1 24H	-13.97059	4.72772	.456	-32.2007	4.2595
	N1 48H	-14.17922	4.72772	.410	-32.4093	4.0509
	N2 24H	-21.34363*	4.72772	.009	-39.5737	-3.1136
	N2 48H	-12.97960	4.72772	.744	-31.2097	5.2505
	N3 24H	-14.30567	4.72772	.385	-32.5357	3.9244
	N3 48H	-17.22092	4.72772	.085	-35.4510	1.0092
	MMS 24H	.00000	4.72772	1.000	-18.2301	18.2301

Dunnett t (2-sided)[b]	AD24H	MMS 48H	13.51100	4.72772	.063	-.5195	27.5415
	AD48H	MMS 48H	2.08333	4.72772	1.000	-11.9472	16.1138
	CS24H	MMS 48H	20.80547*	4.72772	.002	6.7750	34.8360
	CS48H	MMS 48H	11.56648	4.72772	.145	-2.4640	25.5970
	N1 24H	MMS 48H	13.97059	4.72772	.051	-.0599	28.0011
	N1 48H	MMS 48H	14.17922*	4.72772	.047	.1487	28.2097
	N2 24H	MMS 48H	21.34363*	4.72772	.001	7.3131	35.3741
	N2 48H	MMS 48H	12.97960	4.72772	.080	-1.0509	27.0101
	N3 24H	MMS 48H	14.30567*	4.72772	.044	.2752	28.3362
	N3 48H	MMS 48H	17.22092*	4.72772	.011	3.1904	31.2514
	MMS 24H	MMS 48H	.00000	4.72772	1.000	-14.0305	14.0305

*. The mean difference is significant at the 0.05 level.

b. Dunnett t-tests treat one group as a control, and compare all other groups against it.

Table S10. Post-hoc pairwise comparisons of telophase index across treatment groups using Bonferroni and Dunnett's tests.

	(I) VAR00010	(J) VAR00010	Mean Difference (I-J)	Std. Error	Sig.	95% Confidence Interval	
						Lower Bound	Upper Bound
Bonferroni	AD24H	AD48H	10.82817	4.72443	1.000	-7.3892	29.0455
		CS24H	2.75451	4.72443	1.000	-15.4629	20.9719
		CS48H	8.92159	4.72443	1.000	-9.2958	27.1390
		N1 24H	1.68199	4.72443	1.000	-16.5354	19.8994
		N1 48H	5.60258	4.72443	1.000	-12.6148	23.8200
		N2 24H	-4.10294	4.72443	1.000	-22.3203	14.1144
		N2 48H	3.61330	4.72443	1.000	-14.6041	21.8307
		N3 24H	12.71496	4.72443	.842	-5.5024	30.9323
		N3 48H	11.67768	4.72443	1.000	-6.5397	29.8951
		MMS 24H	18.19008	4.72443	.051	-.0273	36.4074
		MMS 48H	18.25674*	4.72443	.049	.0394	36.4741
	AD48H	AD24H	-10.82817	4.72443	1.000	-29.0455	7.3892
		CS24H	-8.07366	4.72443	1.000	-26.2910	10.1437
		CS48H	-1.90658	4.72443	1.000	-20.1240	16.3108
		N1 24H	-9.14618	4.72443	1.000	-27.3636	9.0712
		N1 48H	-5.22559	4.72443	1.000	-23.4430	12.9918
		N2 24H	-14.93111	4.72443	.279	-33.1485	3.2863
		N2 48H	-7.21487	4.72443	1.000	-25.4322	11.0025
		N3 24H	1.88679	4.72443	1.000	-16.3306	20.1042
		N3 48H	.84951	4.72443	1.000	-17.3679	19.0669
		MMS 24H	7.36190	4.72443	1.000	-10.8555	25.5793
		MMS 48H	7.42857	4.72443	1.000	-10.7888	25.6459
	CS24H	AD24H	-2.75451	4.72443	1.000	-20.9719	15.4629
		AD48H	8.07366	4.72443	1.000	-10.1437	26.2910
		CS48H	6.16707	4.72443	1.000	-12.0503	24.3844
		N1 24H	-1.07253	4.72443	1.000	-19.2899	17.1448
		N1 48H	2.84807	4.72443	1.000	-15.3693	21.0654
		N2 24H	-6.85745	4.72443	1.000	-25.0748	11.3599
		N2 48H	.85879	4.72443	1.000	-17.3586	19.0762
		N3 24H	9.96045	4.72443	1.000	-8.2569	28.1778
		N3 48H	8.92317	4.72443	1.000	-9.2942	27.1405
		MMS 24H	15.43556	4.72443	.215	-2.7818	33.6529
		MMS 48H	15.50223	4.72443	.208	-2.7151	33.7196

	CS48H	AD24H	-8.92159	4.72443	1.000	-27.1390	9.2958
		AD48H	1.90658	4.72443	1.000	-16.3108	20.1240
		CS24H	-6.16707	4.72443	1.000	-24.3844	12.0503
		N1 24H	-7.23960	4.72443	1.000	-25.4570	10.9778
		N1 48H	-3.31901	4.72443	1.000	-21.5364	14.8984
		N2 24H	-13.02453	4.72443	.724	-31.2419	5.1928
		N2 48H	-5.30828	4.72443	1.000	-23.5257	12.9091
		N3 24H	3.79337	4.72443	1.000	-14.4240	22.0107
		N3 48H	2.75609	4.72443	1.000	-15.4613	20.9735
		MMS 24H	9.26849	4.72443	1.000	-8.9489	27.4859
		MMS 48H	9.33515	4.72443	1.000	-8.8822	27.5525
	N1 24H	AD24H	-1.68199	4.72443	1.000	-19.8994	16.5354
		AD48H	9.14618	4.72443	1.000	-9.0712	27.3636
		CS24H	1.07253	4.72443	1.000	-17.1448	19.2899
		CS48H	7.23960	4.72443	1.000	-10.9778	25.4570
		N1 48H	3.92059	4.72443	1.000	-14.2968	22.1380
		N2 24H	-5.78493	4.72443	1.000	-24.0023	12.4324
		N2 48H	1.93132	4.72443	1.000	-16.2861	20.1487
		N3 24H	11.03297	4.72443	1.000	-7.1844	29.2503
		N3 48H	9.99569	4.72443	1.000	-8.2217	28.2131
		MMS 24H	16.50809	4.72443	.123	-1.7093	34.7255
		MMS 48H	16.57475	4.72443	.119	-1.6426	34.7921
	N1 48H	AD24H	-5.60258	4.72443	1.000	-23.8200	12.6148
		AD48H	5.22559	4.72443	1.000	-12.9918	23.4430
		CS24H	-2.84807	4.72443	1.000	-21.0654	15.3693
		CS48H	3.31901	4.72443	1.000	-14.8984	21.5364
		N1 24H	-3.92059	4.72443	1.000	-22.1380	14.2968
		N2 24H	-9.70552	4.72443	1.000	-27.9229	8.5119
		N2 48H	-1.98928	4.72443	1.000	-20.2066	16.2281
		N3 24H	7.11238	4.72443	1.000	-11.1050	25.3298
		N3 48H	6.07510	4.72443	1.000	-12.1423	24.2925
		MMS 24H	12.58750	4.72443	.896	-5.6299	30.8049
		MMS 48H	12.65416	4.72443	.867	-5.5632	30.8715
	N2 24H	AD24H	4.10294	4.72443	1.000	-14.1144	22.3203
		AD48H	14.93111	4.72443	.279	-3.2863	33.1485
		CS24H	6.85745	4.72443	1.000	-11.3599	25.0748
		CS48H	13.02453	4.72443	.724	-5.1928	31.2419
		N1 24H	5.78493	4.72443	1.000	-12.4324	24.0023
		N1 48H	9.70552	4.72443	1.000	-8.5119	27.9229
		N2 48H	7.71624	4.72443	1.000	-10.5011	25.9336
		N3 24H	16.81790	4.72443	.105	-1.3995	35.0353
		N3 48H	15.78062	4.72443	.180	-2.4368	33.9980
		MMS 24H	22.29302*	4.72443	.006	4.0756	40.5104
		MMS 48H	22.35968*	4.72443	.005	4.1423	40.5771
	N2 48H	AD24H	-3.61330	4.72443	1.000	-21.8307	14.6041
		AD48H	7.21487	4.72443	1.000	-11.0025	25.4322
		CS24H	-.85879	4.72443	1.000	-19.0762	17.3586
		CS48H	5.30828	4.72443	1.000	-12.9091	23.5257
		N1 24H	-1.93132	4.72443	1.000	-20.1487	16.2861
		N1 48H	1.98928	4.72443	1.000	-16.2281	20.2066
		N2 24H	-7.71624	4.72443	1.000	-25.9336	10.5011
		N3 24H	9.10166	4.72443	1.000	-9.1157	27.3190
		N3 48H	8.06438	4.72443	1.000	-10.1530	26.2817
		MMS 24H	14.57677	4.72443	.334	-3.6406	32.7941
		MMS 48H	14.64344	4.72443	.323	-3.5739	32.8608

	N3 24H	AD24H	-12.71496	4.72443	.842	-30.9323	5.5024
		AD48H	-1.88679	4.72443	1.000	-20.1042	16.3306
		CS24H	-9.96045	4.72443	1.000	-28.1778	8.2569
		CS48H	-3.79337	4.72443	1.000	-22.0107	14.4240
		N1 24H	-11.03297	4.72443	1.000	-29.2503	7.1844
		N1 48H	-7.11238	4.72443	1.000	-25.3298	11.1050
		N2 24H	-16.81790	4.72443	.105	-35.0353	1.3995
		N2 48H	-9.10166	4.72443	1.000	-27.3190	9.1157
		N3 48H	-1.03728	4.72443	1.000	-19.2547	17.1801
		MMS 24H	5.47512	4.72443	1.000	-12.7423	23.6925
		MMS 48H	5.54178	4.72443	1.000	-12.6756	23.7592
	N3 48H	AD24H	-11.67768	4.72443	1.000	-29.8951	6.5397
		AD48H	-.84951	4.72443	1.000	-19.0669	17.3679
		CS24H	-8.92317	4.72443	1.000	-27.1405	9.2942
		CS48H	-2.75609	4.72443	1.000	-20.9735	15.4613
		N1 24H	-9.99569	4.72443	1.000	-28.2131	8.2217
		N1 48H	-6.07510	4.72443	1.000	-24.2925	12.1423
		N2 24H	-15.78062	4.72443	.180	-33.9980	2.4368
		N2 48H	-8.06438	4.72443	1.000	-26.2817	10.1530
		N3 24H	1.03728	4.72443	1.000	-17.1801	19.2547
		MMS 24H	6.51240	4.72443	1.000	-11.7050	24.7298
		MMS 48H	6.57906	4.72443	1.000	-11.6383	24.7964
	MMS 24H	AD24H	-18.19008	4.72443	.051	-36.4074	.0273
		AD48H	-7.36190	4.72443	1.000	-25.5793	10.8555
		CS24H	-15.43556	4.72443	.215	-33.6529	2.7818
		CS48H	-9.26849	4.72443	1.000	-27.4859	8.9489
		N1 24H	-16.50809	4.72443	.123	-34.7255	1.7093
		N1 48H	-12.58750	4.72443	.896	-30.8049	5.6299
		N2 24H	-22.29302*	4.72443	.006	-40.5104	-4.0756
		N2 48H	-14.57677	4.72443	.334	-32.7941	3.6406
		N3 24H	-5.47512	4.72443	1.000	-23.6925	12.7423
		N3 48H	-6.51240	4.72443	1.000	-24.7298	11.7050
		MMS 48H	.06667	4.72443	1.000	-18.1507	18.2840
	MMS 48H	AD24H	-18.25674*	4.72443	.049	-36.4741	-.0394
		AD48H	-7.42857	4.72443	1.000	-25.6459	10.7888
		CS24H	-15.50223	4.72443	.208	-33.7196	2.7151
		CS48H	-9.33515	4.72443	1.000	-27.5525	8.8822
		N1 24H	-16.57475	4.72443	.119	-34.7921	1.6426
		N1 48H	-12.65416	4.72443	.867	-30.8715	5.5632
		N2 24H	-22.35968*	4.72443	.005	-40.5771	-4.1423
		N2 48H	-14.64344	4.72443	.323	-32.8608	3.5739
		N3 24H	-5.54178	4.72443	1.000	-23.7592	12.6756
		N3 48H	-6.57906	4.72443	1.000	-24.7964	11.6383
		MMS 24H	-.06667	4.72443	1.000	-18.2840	18.1507
Dunnett t (2-sided)[b]	AD24H	MMS 48H	18.25674*	4.72443	.006	4.2360	32.2775
	AD48H	MMS 48H	7.42857	4.72443	.583	-6.5921	21.4493
	CS24H	MMS 48H	15.50223*	4.72443	.025	1.4815	29.5229
	CS48H	MMS 48H	9.33515	4.72443	.331	-4.6856	23.3559
	N1 24H	MMS 48H	16.57475*	4.72443	.015	2.5540	30.5955
	N1 48H	MMS 48H	12.65416	4.72443	.092	-1.3665	26.6749
	N2 24H	MMS 48H	22.35968*	4.72443	.001	8.3390	36.3804
	N2 48H	MMS 48H	14.64344*	4.72443	.037	.6227	28.6642
	N3 24H	MMS 48H	5.54178	4.72443	.849	-8.4789	19.5625
	N3 48H	MMS 48H	6.57906	4.72443	.709	-7.4417	20.5998
	MMS 24H	MMS 48H	.06667	4.72443	1.000	-13.9540	14.0874

*. The mean difference is significant at the 0.05 level.
b. Dunnett t-tests treat one group as a control, and compare all other groups against it.

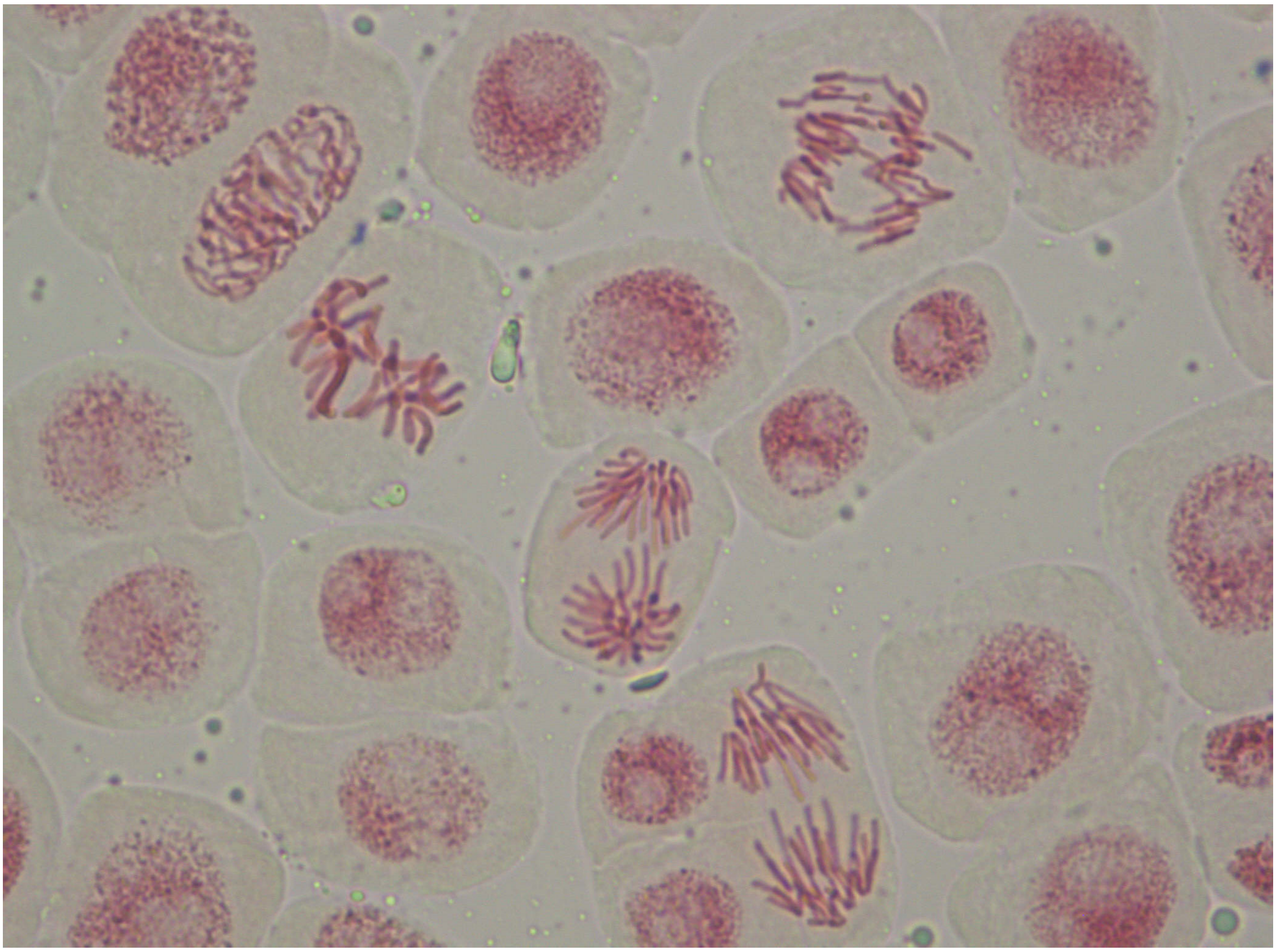

Figure S3. Chromosomal aberrations identified in root meristematic cells of A. cepa that underwent treatment with silver nanoparticles (20 nm, 0.02 mg/mL dispersed in a 2 mM sodium citrate solution, λmax: 405 nm): aAnaphase bridges, N2 24H.

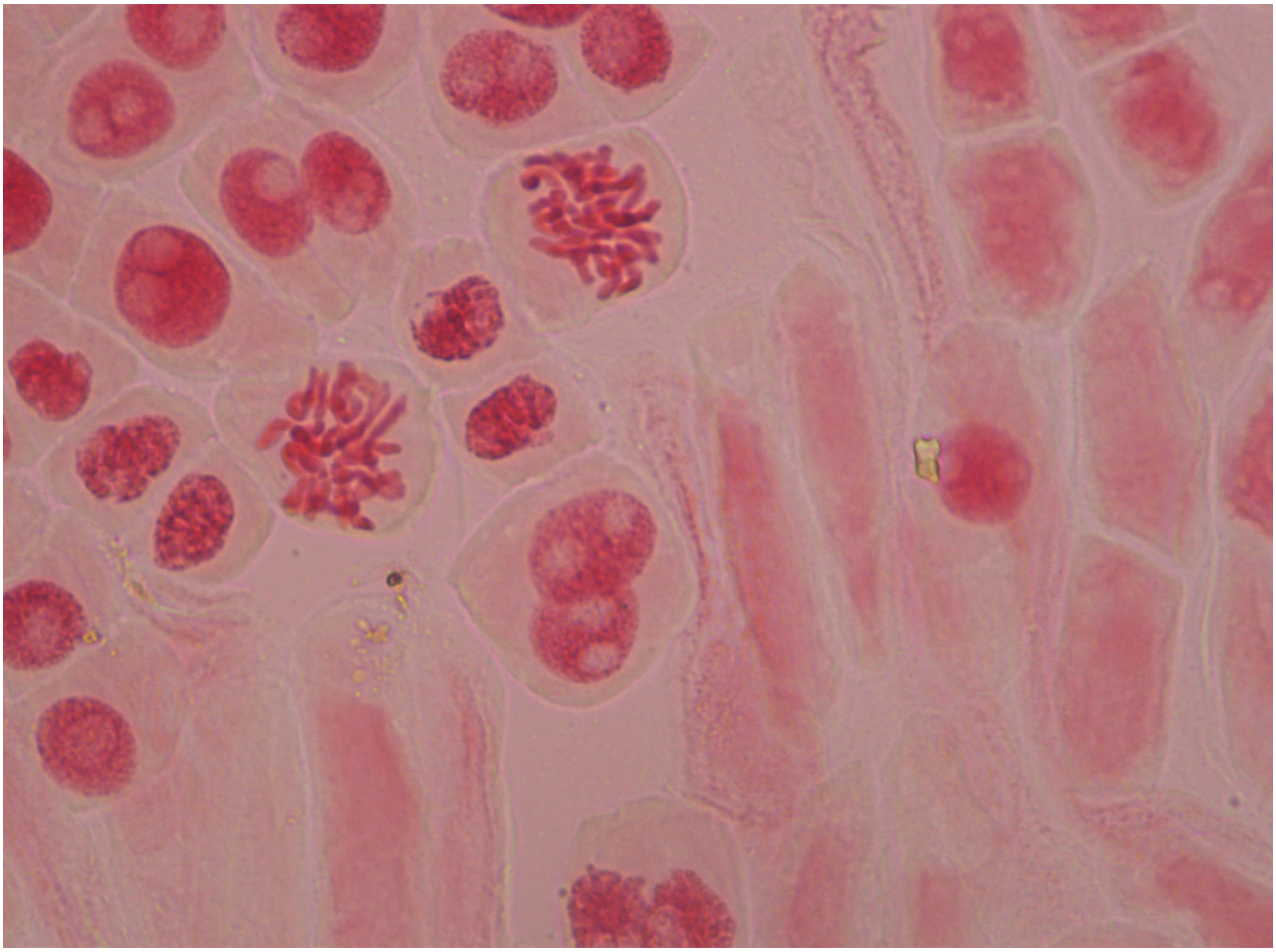

Figure S4. Chromosomal aberrations identified in root meristematic cells of A. cepa that underwent treatment with silver nanoparticles (20 nm, 0.02 mg/mL dispersed in a 2 mM sodium citrate solution, λmax: 405 nm): C-mitosis, N1 24H.

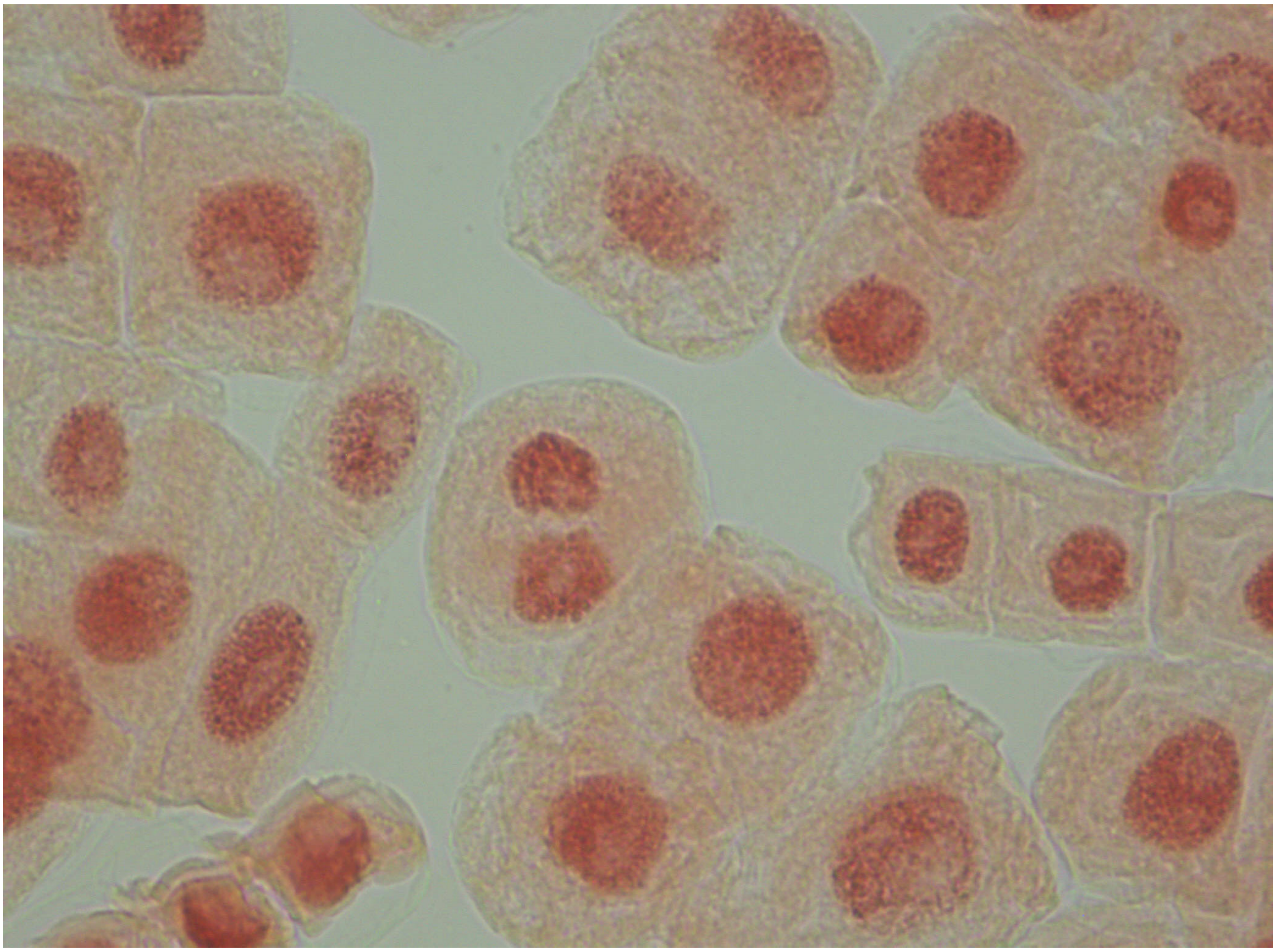

Figure S5. Chromosomal aberrations identified in root meristematic cells of A. cepa that underwent treatment with silver nanoparticles (20 nm, 0.02 mg/mL dispersed in a 2 mM sodium citrate solution, λmax: 405 nm): binucleate cell, N1 48H.

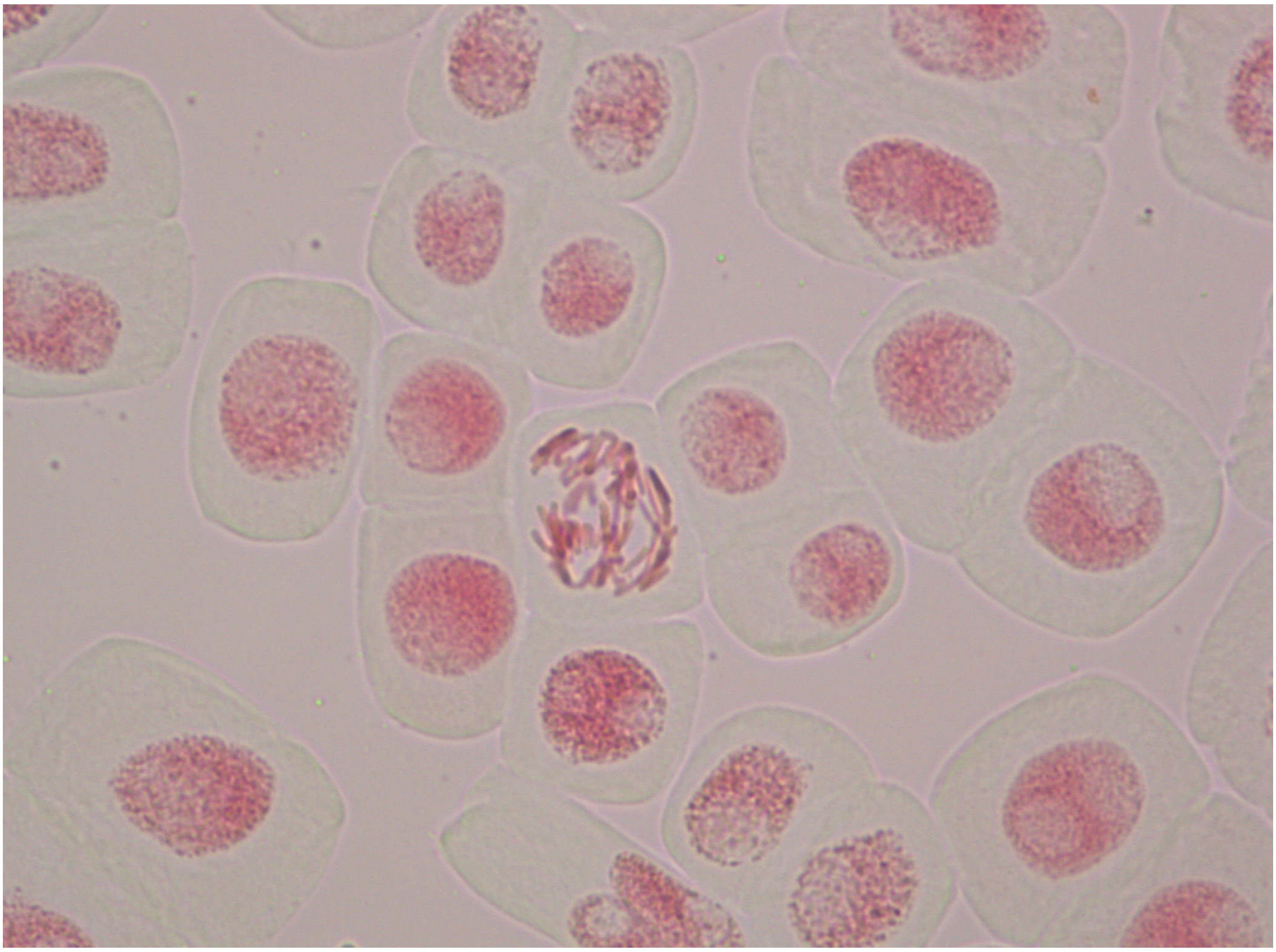

Figure S6. Chromosomal aberrations identified in root meristematic cells of A. cepa that underwent treatment with silver nanoparticles (20 nm, 0.02 mg/mL dispersed in a 2 mM sodium citrate solution, λmax: 405 nm): multipolar anaphase anaphases showing chromosomes bridges—N2 24H.

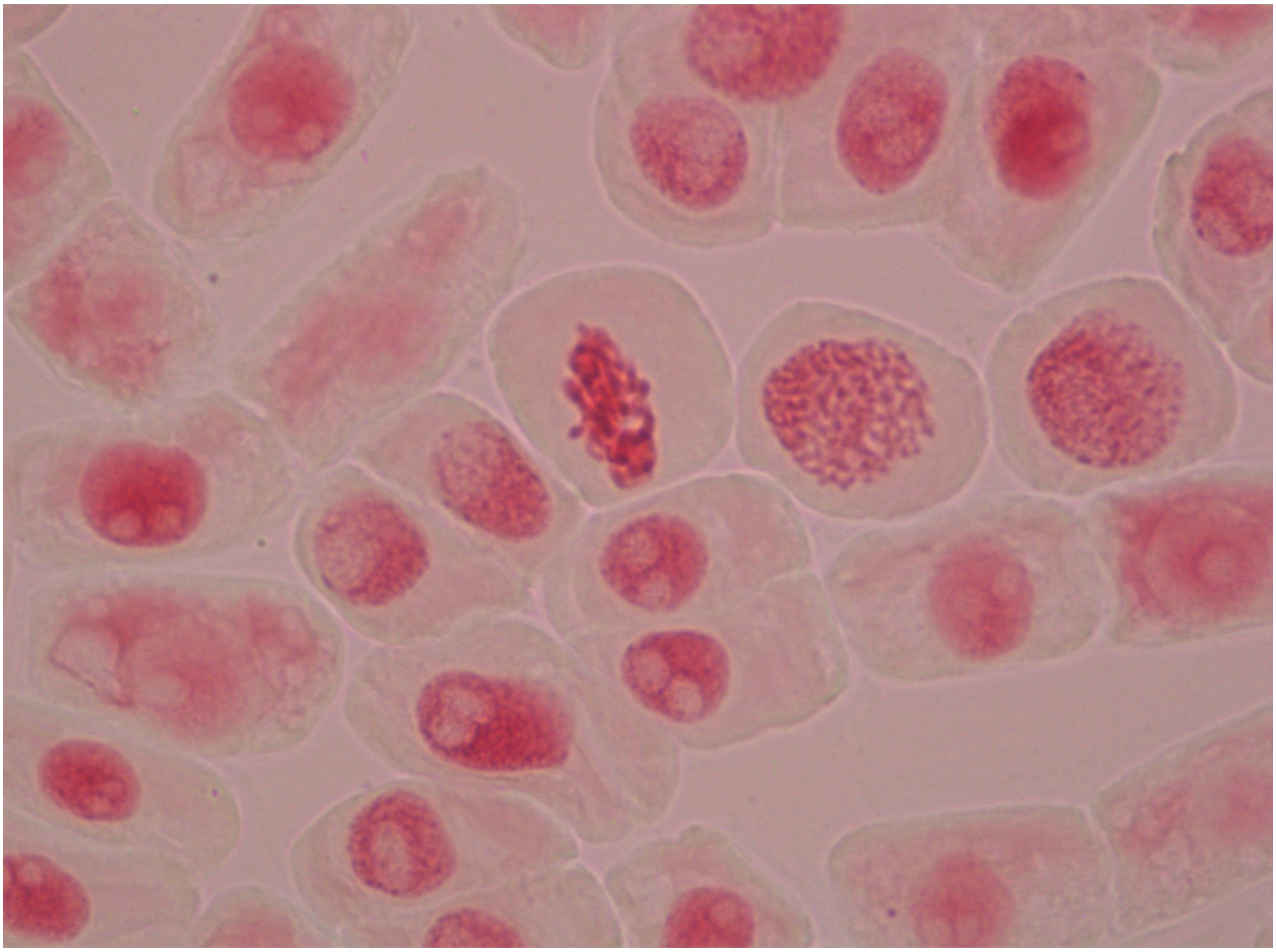

Figure. S7. Chromosomal aberrations identified in root meristematic cells of A. cepa that underwent treatment with silver nanoparticles (20 nm, 0.02 mg/mL dispersed in a 2 mM sodium citrate solution, λmax: 405 nm): sticky chromosomes, N3 48S.

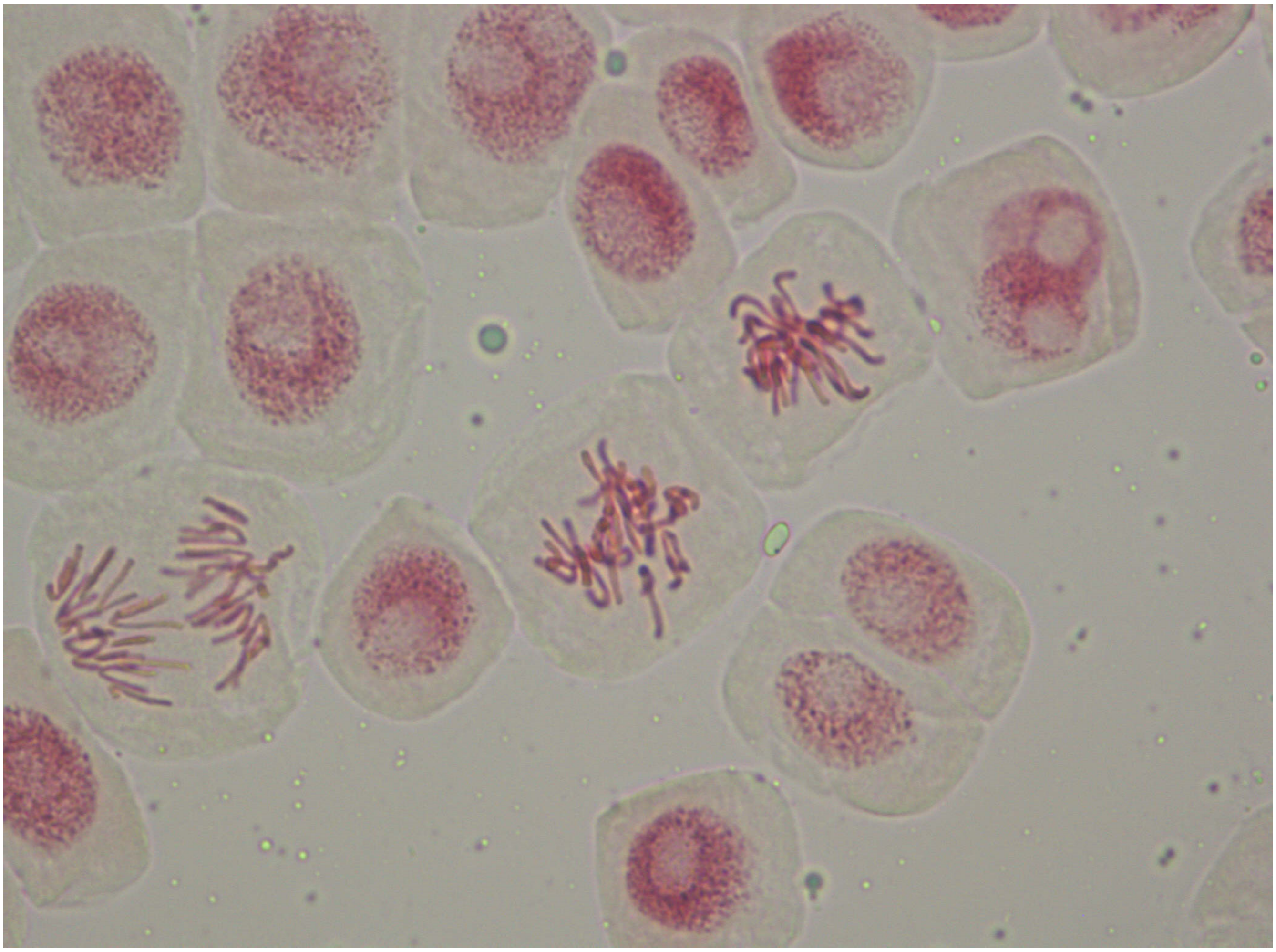

Figure S8. Chromosomal aberrations identified in root meristematic cells of A. cepa that underwent treatment with silver nanoparticles (20 nm, 0.02 mg/mL dispersed in a 2 mM sodium citrate solution, λmax: 405 nm): lagging chromosomes, N1 48H.
